# Efficacy and safety of Duhuo-Jisheng decoction in rheumatoid arthritis: A systematic review and meta-analysis of 42 randomized controlled trials

**DOI:** 10.1097/MD.0000000000035513

**Published:** 2023-11-03

**Authors:** Pengda Qu, Haiyang Wang, Wei Wang, Shiyu Du, Zhaorong Peng, Qian Hu, Xiaohu Tang

**Affiliations:** a First Clinical Medical College, Yunnan University of Chinese Medicine, Kunming, China; b School of Basic Medical Sciences, Yunnan University of Chinese Medicine, Kunming, China; c Department of Rheumatology, Yunnan Provincial Hospital of Traditional Chinese Medicine, Kunming, China.

**Keywords:** Duhuo-Jisheng decoction, meta-analysis, rheumatoid arthritis, systematic review, traditional Chinese medicine

## Abstract

**Background:**

Duhuo-Jisheng decoction (DJD) is a Chinese herb formula. Previous studies have reported that the clinical symptoms and laboratory indicators of rheumatoid arthritis (RA) patients could be improved by DJD. However, the existing evidence was not robust enough and controversial.

**Methods:**

Randomized controlled trials of DJD for RA were retrieved from Chinese and English databases from their inception to April 16, 2023. Meta-analysis was performed by Stata 17 software. We used subgroup analysis, meta-regression, and sensitivity analysis to identify potential sources of heterogeneity. The subgroup analysis and meta-regression were conducted from 6 aspects, including age, course of disease, course of treatment, interventions used in the experimental or control group, and random sequence generation. Galbraith plot was used to find studies with possible heterogeneity. Publication bias was assessed by Egger’s test and funnel plots when the number of relevant studies was greater than or equal to 10.

**Results:**

Forty-two studies were included, involving 3635 patients and 19 outcome indicators. Meta-analysis showed that, compared with the routine disease-modifying antirheumatic drugs (rDMARDs), DJD could better improve the level of laboratory indicators, main symptoms and signs, and questionnaire scores of RA patients. The laboratory indicators included rheumatoid factor, T lymphocyte subpopulation (including CD4^+^, CD8^+^, and CD4^+^/CD8^+^), and inflammatory biomarkers (including erythrocyte sedimentation rate, C-reactive protein, tumor necrosis factor-α, interleukin 6, interleukin 1β, and interleukin 1). The main symptoms and signs included the duration of morning stiffness, the number of joint tenderness, the number of swollen joints, and the grip strength of both hands. The questionnaire included visual analogue scale, health assessment questionnaire, and disease activity score in 28 joints. In addition, the adverse events of DJD treatment were significantly lower than those of rDMARDs. However, the results of a few subgroup analyses differed from the overall results. Furthermore, the publication bias assessment showed that, out of 11 evaluated results, 4 had publication bias.

**Conclusion:**

DJD could be a satisfactory complementary and alternative therapy for RA. However, due to a small number of subgroup analysis results being different from the overall results, it should be verified by further studies.

## 1. Introduction

Rheumatoid arthritis (RA) is a chronic, systemic autoimmune disease characterized clinically by erosive, symmetrical polyarthritis.^[1]^ Its basic pathology is manifested by the formation of synovitis and pannus, and the destruction of articular cartilage and bone occurs, ultimately leading to joint deformity and loss of function, which leads to productivity loss, disability, as well as heavy financial burden.^[1,2]^ In addition, it was also a risk factor for osteoporosis, cardiovascular, etc.^[3,4]^ The worldwide prevalence of RA was estimated to be 0.46%.^[5]^ In China, the prevalence was approximately 0.20% to 0.37%.^[6]^ Therefore, studies on the treatment of RA have been paid attention to by rheumatologists. Currently, medications used to treat RA include methotrexate, leflunomide, meloxicam, glucocorticoids, etc., according to 2022 updated EULAR recommendations for the management of rheumatoid arthritis,^[7]^ and the 2021 American College of Rheumatology guideline.^[8]^ Although the use of the above drugs has achieved relatively satisfactory clinical efficacy, their adverse reactions cannot be ignored. For example, glucocorticoids could induce an increased risk of osteoporotic fractures, serious infections, diabetes, and mortality^[9]^; disease-modifying antirheumatic drugs may induce malignancies and major adverse cardiovascular events more frequently.^[10]^ Consequently, it is necessary to find alternative strategies to treat RA more safely and effectively.

In China, traditional Chinese medicine (TCM) has been considered an important alternative therapy to treat RA. Because it has been used to treat rheumatic diseases for thousands of years, which has accumulated a lot of experience. Duhuo-Jisheng decoction (DJD) is a formula of TCM, which is composed of 15 herbs, including *Radix Angelicae Pubescentis* (Du Huo), *Herba Taxilli* (Sang Ji Sheng), *Cortex Eucommiae* (Du Zhong), *Radix Achyranthis bidentatae* (Niu Xi), *Herba Asari* (Xi Xin), *Radix Gentiae macrophyllae* (Qin Jiao), *Poria* (Fu Ling), *Cortex Cinmomi* (Rou Gui), *Radix Saposhnikoviae divaricatae* (Fang Feng), *Radix chuanxiong* (Chuan Xiong), *Radix Ginseng* (Ren Shen), *Radix Glycyrrhizae* (Gan Cao), *Radix Angelicae sinensis* (Dang Gui), *Radix Paeoniae Alba* (Bai Shao), and *Rehmannia glutinosa* (Gan Di Huang). In TCM theory, DJD has the effect of dispelling wind and dampness, relieving arthralgia and pain, benefiting the liver and kidneys, and replenishing qi and blood, which is closely related to the etiology and pathogenesis of RA. Previous studies reported that the clinical symptoms and laboratory indicators of RA patients could be significantly improved by DJD, including the duration of morning stiffness, the number of joint tenderness, the level of erythrocyte sedimentation rate, C-reactive protein, and rheumatoid factor.^[11–14]^ In addition, many researchers had argued that DJD therapy had fewer adverse reactions than routine disease-modifying antirheumatic drugs (rDMARDs).^[15–18]^ Accordingly, DJD is considered an important complementary and alternative therapy for the treatment of RA. However, there are few evidence-based conclusions due to the small sample sizes and some controversial results of available clinical trials. Therefore, it is significant to evaluate the efficacy and safety of DJD in the treatment of RA. In this study, we evaluated the efficacy and safety of DJD in patients with RA by meta-analysis to provide clinicians with robust evidence-based evidence for the management of RA.

## 2. Materials and Methods

### 2.1. Protocol registration

This meta-analysis was conducted and reported according to the guidelines of Preferred Reporting Items for Systematic Reviews and Meta-Analyses (PRISMA).^[19]^ Research protocol (No. CRD42023410621) was registered in the PROSPERO (https://www.crd.york.ac.uk/PROSPERO/).

### 2.2. Database and search strategy

The databases included Web of Science, PubMed, Cochrane, Embase, Wanfang database, China national knowledge infrastructure (CNKI), and China Science and Technology Journal Database (VIP). Studies were searched from the establishment of the database until April 16, 2023, with language limited to Chinese or English. The search terms were MeSH terms combined with the keywords “Duhuo-Jisheng decoction” and “rheumatoid arthritis.” Article references were further searched for additional relevant publications. The specific search strategy of PubMed was shown in Table S1 (see Table S1, Supplemental Digital Content, http://links.lww.com/MD/K288).

### 2.3. Inclusion and exclusion criteria

The inclusion criteria were as follows:

1. Research subjects were diagnosed with RA according to the diagnostic criteria of the 1987 American Rheumatology Association guidelines or the 2010 ACR/European League Against Rheumatism (EULAR) criteria.^[20,21]^
2. The experimental group was treated with DJD or DJD combined with the medications of routine treatment, such as methotrexate, leflunomide, and meloxicam.
3. The control group was treated with the medications of rDMARDs only.
4. The outcome indicators included effective rate, erythrocyte sedimentation rate (ESR), C-reactive protein (CRP), rheumatoid factor (RF), tumor necrosis factor-α (TNF-α), interleukin 1 (IL-1), interleukin 1β (IL-1β), interleukin 6 (IL-6), T lymphocyte subpopulation (including CD4+, CD8+, and CD4+/CD8+), duration of morning stiffness, number of joint tenderness, number of swollen joints, grip strength of both hands, visual analogue scale (VAS), health assessment questionnaire (HAQ), disease activity score in 28 joints (DAS-28), or adverse events (AEs). Primary outcome indicators were ESR, CRP, RF, TNF-α, IL-1, IL-1β, IL-6, T lymphocyte subpopulation (including CD4+, CD8+, and CD4+/CD8+), and AEs. Secondary outcome indicators were effective rate, duration of morning stiffness, number of joint tenderness, number of swollen joints, grip strength of both hands, VAS, HAQ, and DAS-28.
5. The type of study was a randomized controlled trial.

The exclusion criteria were as follows: Experimental group with acupuncture, tuina, acupoint application, or other external therapies of TCM, Control group with any other therapies of TCM, Duplicate publications, Studies with incomplete data, and Unable to extract data for research.

### 2.4. Data extraction

After excluding irrelevant studies by reading titles and abstracts, 2 independent reviewers collected data from the included articles using a pre-designed form. The extracted information included: the investigator, year of publication, number of cases, age, intervention measures, duration of intervention, outcome indicators, and adverse events. Any controversy was resolved by discussion with XT.

### 2.5. Bias assessment

The methodological quality of the included studies was evaluated independently by 2 reviewers using the Cochrane Collaboration’s tool.^[22]^ Three levels, low risk, high risk, and unclear risk, were used to evaluate 7 items: random sequence generation, allocation concealment, blind method, blind evaluation of results, incomplete result data, selective reporting, and other biases. Any disagreements were resolved by discussion with XT.

### 2.6. Statistical analysis

Meta-analysis was carried out by Stata 17 software (Stata Corp., College Station, TX). Weighted mean difference (WMD) or standardized mean difference was used to represent continuous variables and risk ratio was used for binary variables. WMD was used when the same measures or units were applied to the same intervention effect. Otherwise, standardized mean difference was used. All variables were expressed with a 95% confidence interval (CI). The heterogeneity was tested by the Q test. The fixed effects model was used when *I*^2^ was less than or equal to 50%. When *I*^2^ was greater than 50%, the random effects model was used, and subgroup analysis, meta-regression, and sensitivity analysis were conducted to identify potential sources of heterogeneity. The subgroup analysis and meta-regression were conducted from 6 aspects, including age, course of disease, course of treatment, interventions used in the experimental or control group, and random sequence generation. Subsequently, Galbraith plots were used to find studies with possible heterogeneity. After these studies were removed, the meta-analysis was re-performed. The results of the meta-analysis were expressed by forest plots. Publication bias was assessed by Egger’s test and funnel plots when the number of relevant studies was greater than or equal to 10.

## 3. Results

### 3.1. Characteristics of eligible studies

**Figure 1.**
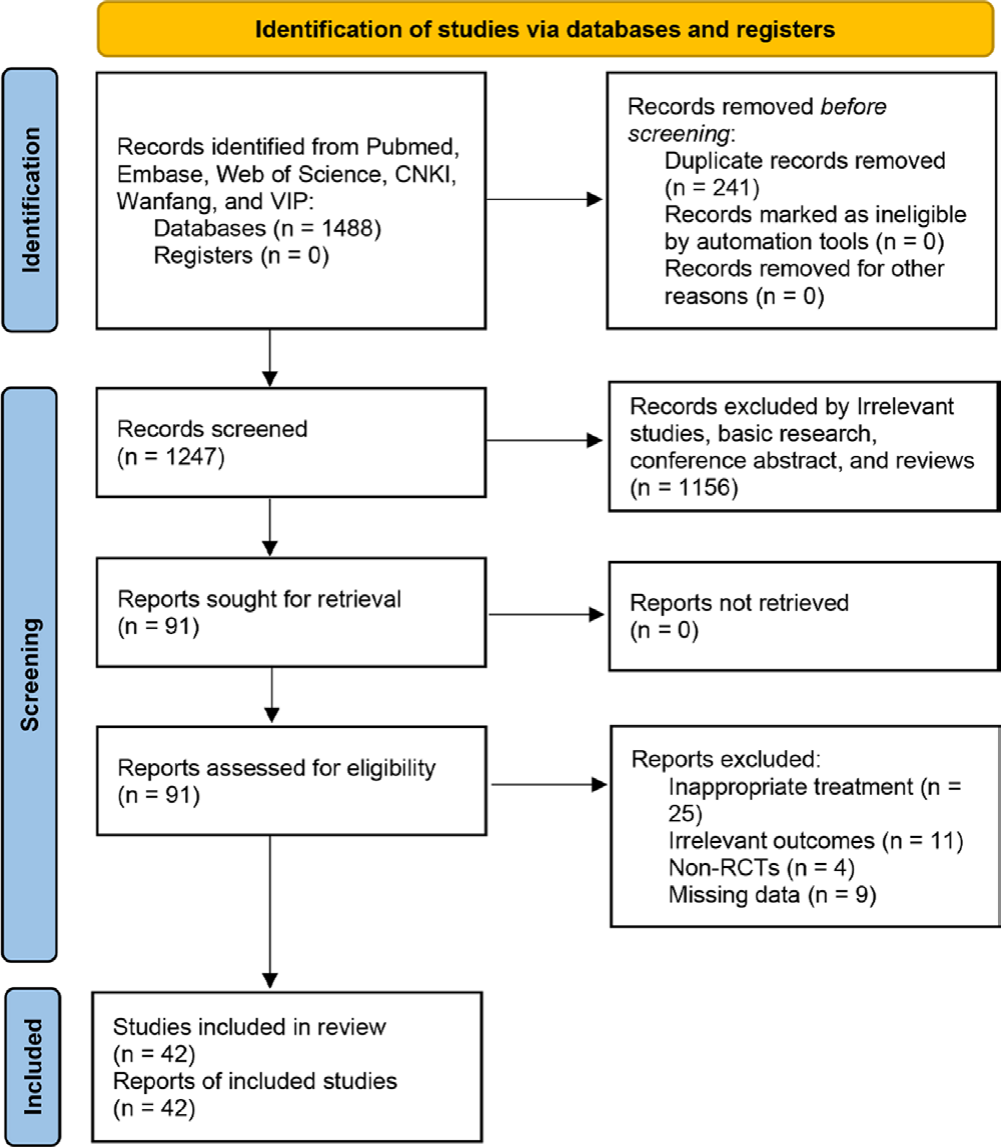
Flow diagram of the study selection.

Forty-two studies were included in our analysis.^[11–18,23–56]^ The screening flow was shown in Figure 1. The 42 trials, published between 2012 and 2022, enrolled 3635 individuals, of whom 1824 were in the experimental group and 1811 were in the control group. Details can be seen in Table 1.

**Table 1:** Characteristics of the included studies.

Study	Sample T(male/female)/Sample C(male/female)	Mean age or age range (T/C)	Course or course range (T/C)	Interventions		Duration	Outcomes
				T	C		
Cao et al (2014)	29(10/19)/29(7/22)	42.6 ± 6.7/41.9 ± 5.5	3.7 ± 2.8 yr/3.6 ± 2.5 yr	DJD	MTX	2 mo	1.2.3.4.12.19.
Cao et al (2020)	41(14/29)/41(13/28)	38.67 ± 9.52/39.11 ± 9.37	2.55 ± 0.69 yr/2.65 ± 0.72 yr	DJD + C	MTX	6 wk	1.2.3.4.12.13.14.15.16.17.
Chen (2017a)	63(26/37)/63(24/39)	37.62 ± 5.27/38.05 ± 6.62	-	DJD + C	Meloxicam	3 mo	1.2.3.4.5.8.12.15.17.19.
Chen et al (2020a)	30(7/23)/30(8/22)	41.6 ± 5.2/42.8 ± 4.7	3.2 yr/3.3 yr	DJD	LEF	8 wk	2.13.14.16.18.
Chen (2019)	25(2/23)/25(4/21)	51.4 ± 5.8/53.6 ± 8.6	8.0 ± 3.9 mo/7.9 ± 4.1 mo	DJD + C	MTX + LEF + Meloxicam	12 wk	1.2.3.4.17.18.19.
Chen et al (2020b)	30(13/17)/30(14/16)	47.14 ± 3.28/48.05 ± 3.42	5.13 ± 2.17 yr/5.25 ± 2.26 yr	DJD + C	MTX + Iguratimod	3 mo	1.5.6.12.13.14.15.17.18.19.
Chen (2017b)	45(-/-)/45(-/-)	52.34 ± 3.26/52.34 ± 3.26	4.28 ± 1.37 yr/4.28 ± 1.37 yr	DJD + C	LEF	3 mo	1.2.3.4.
Deng and Xie (2022)	41(10/31)/41(9/32)	40.5 ± 4.2/40.1 ± 4.5	3.1 ± 0.5 yr/3.3 ± 0.4 yr	DJD + C	MTX + Diclofenac	2 mo	1.2.3.4.5.6.16.18.19.
Ding et al (2021)	40(28/12)/40(26/14)	43.2 ± 12.4/42.3 ± 11.2	6.28 ± 2.20 yr/5.82 ± 2.30 yr	DJD + C	Iguratimod + HCQ	3 mo	1.5.6.
Gong (2022)	42(22/20)/42(19/23)	58.33 ± 8.41/60.38 ± 10.19	8.05 ± 2.49 yr/8.45 ± 2.18 yr	DJD	Celecoxib	2 wk	1.5.6.7.19.
Guo and Wang (2018)	46(15/31)/46(17/29)	62.83 ± 3.94/63.07 ± 3.11	14.97 ± 2.86 mo/15.02 ± 2.13 mo	DJD + C	LEF + MTX	3 mo	1.2.3.4.5.6.8.12.13.14.15.
He and Li (2021)	49(32/17)/49(31/18)	44.8 ± 5.2/43.7 ± 4.9	-	DJD + C	Diclofenac	12 wk	1.9.10.11.17.19.
Huang (2012)	35(1718/)/35(15/20)	51.2 ± 1.2/54.1 ± 1.3	3.2 ± 1.3 yr/3.3 ± 1.2 yr	DJD + C	Meloxicam + MTX	4 wk	1.2.3.4.12.13.14.15.16.
Ji (2015)	78(-/-)/77(-/-)	43.8 ± 17.9/41.8 ± 18.6	-	DJD + C	LEF	12 wk	1.2.3.12.19.
Jiang (2014)	40(21/19)/40(18/22)	46.1 ± 9.3/46.1 ± 9.3	4.6 ± 1.2 yr/4.6 ± 1.2 yr	DJD	Diclofenac	12 wk	1.2.3.12.
Li et al (2012)	30(14/16)/30(17/13)	45.16 ± 6.48/44.86 ± 6.23	2.26 ± 0.75 yr/2.32 ± 0.83 yr	DJD + C	SSZ	3 mo	1.2.3.4.12.13.14.19.
Li and Liu (2017)	30(5/25)/30(5/25)	66 ± 3.1/64 ± 2.0	20 ± 8.1 mo/20 ± 8.5 mo	DJD + C	LEF	12 wk	1.2.3.4.12.13.14.
Li (2021)	40(6/34)/40(8/32)	61/60	5.6 ± 2.1 yr/5.3 ± 2.4 yr	DJD + C	MTX	3 mo	1.2.3.4.17.19.
Mei et al (2020)	54(21/33)/54(20/34)	52.23 ± 8.79/54.08 ± 8.68	10.40 ± 5.85 yr/10.80 ± 6.89 yr	DJD + C	LEF + Iron Dextran + Folic Acid	8 wk	4.18.19.
Ni et al (2015)	38(21/17)/38(18/20)	36.2 ± 6.5/37.5 ± 5.9	6.3 ± 2.9 yr/7.1 ± 2.6 yr	DJD + C	MTX	3 mo	1.2.3.4.5.6.19.
Qian et al (2016)	47(20/27)/45(19/26)	56.57 ± 8.59/58.25 ± 9.01	3.61 ± 0.98 yr/3.74 ± 1.09 yr	DJD + C	Diclofenac + MTX	3 mo	1.2.3.4.5.8.12.13.14.15.17.
Qiu et al (2018)	30(3/27)/30(2/28)	45.7 ± 12.6/46.4 ± 14.2	4.9 ± 1.1 yr/4.3 ± 0.8 yr	DJD + C	Meloxicam	12 wk	1.2.3.4.12.13.14.
Shan (2018)	51(28/26)/51(27/24)	48.47 ± 6.54/49.82 ± 5.84	7.42 ± 5.37 yr/6.84 ± 6.34 yr	DJD + C	MTX + HCQ + Meloxicam	6 mo	1.9.10.11.12.14.
Shi et al (2019)	40(15/25)/40(15/25)	36.5 ± 1.2/35.5 ± 2.7	6.5 ± 1.2 yr/4.5 ± 0.7 yr	DJD + C	Meloxicam + MTX	3 mo	1.12.16.18.
Song and Dai (2016)	60(33/27)/60(31/29)	55.3 ± 6.4/56.9 ± 5.9	2.9 ± 0.6 yr/3.1 ± 0.5 yr	DJD + C	MTX	1 mo	1.2.3.4.12.16.19.
Wan et al (2020)	39(23/16)/39(24/15)	55.73/56.83	6.22 ± 1.48 yr/6.82 ± 1.31 yr	DJD	MTX + Celecoxib	2 mo	1.12.13.14.15.
Wang and Qian (2017)	35(16/19)/35(14/21)	31.4 ± 15.7/30.5 ± 17.2	5.8 ± 1.7 yr/5.3 ± 1.8 yr	DJD + C	Ripson + Ibuprofen	2 mo	1.2.3.12.13.14.16.17.19.
Wang et al (2019)	43(14/29)/43(16/27)	54.8 ± 6.2/56.5 ± 6.7	6.6 ± 1.2 mo/6.8 ± 1.3 mo	DJD	Tripterygium Glycosides	1 mo	1.3.6.18.
Wang and Liu (2015)	50(16/34)/50(19/31)	24.5 ± 3.5/24.9 ± 4.5	4.3 ± 0.9 yr/4.4 ± 0.8 yr	DJD + C	MTX + SSZ	3 mo	1.2.3.4.18.19.
Wei and Jia (2021)	52(19/33)/52(22/30)	49.4 ± 5.6/49.2 ± 5.7	22.2 ± 3.3 mo/22.4 ± 3.5 mo	DJD + C	Ibuprofen + LEF	4 wk	1.12.13.14.
Xu and Ji (2022)	100(41/59)/100(42/58)	47.48 ± 3.22/47.53 ± 3.17	25.05 ± 1.17 mo/24.96 ± 1.14 mo	DJD + C	Meloxicam	4 wk	1.2.4.5.6.7.19.
Yang et al (2020)	40(18/22)/40(21/19)	55.23 ± 3.35/54.44 ± 3.31	3.23 ± 1.12 yr/3.21 ± 1.11 yr	DJD + C	Meloxicam + Ibuprofen + Celecoxib + MTX	4 wk	1.2.3.4.9.10.11.12.19.
Yao (2013)	30(3/27)/30(5/25)	45.17 ± 10.79/47.42 ± 11.47	3.98 ± 2.35 yr/4.12 ± 2.56 yr	DJD + C	MTX + Celebrex	2 mo	1.2.3.4.16.19.
Yu et al (2014)	46(12/34)/36(9/27)	32.28 ± 5.17/31.16 ± 4.84	4.4 yr/4.2 yr	DJD + C	LEF + Nimesulide	6 mo	1.2.3.4.12.13.15.19.
Yu and Chen (2015)	50(-/-)/50(-/-)	47.2 ± 4.8/47.2 ± 4.8	4.3 ± 2.1 yr/4.3 ± 2.1 yr	DJD + C	Meloxicam	3 mo	1.2.3.4.5.6.7.12.19.
Zhang (2019)	56(31/25)/56(32/24)	46.5 ± 6.4/47.5 ± 6.5	6.2 ± 1.4 yr/5.9 ± 1.5 yr	DJD + C	MTX	2 mo	1.3.5.6.12.13.14.19.
Zhang et al (2013)	30(9/21)/30(8/22)	13.6 ± 5.3/42.5 ± 4.7	3.6 yr/3.6 yr	DJD + C	LEF	16 wk	1.2.3.4.12.13.14.16.
Zhang and Wang (2018)	35(16/19)/35(14/21)	40.2 ± 14.3/39.5 ± 16.2	6.3 ± 1.4 yr/5.4 ± 1.8 yr	DJD	Ibuprofen + MTX	2 mo	1.
Zhang et al (2019b)	51(18/33/)/51(20/31)	70.12 ± 4.42/69.94 ± 3.81	4.08 ± 0.94 yr/4.17 ± 0.82 yr	DJD + C	LEF	3 mo	1.2.4.5.6.7.13.14.
Zhao et al (2021a)	24(6/18)/24(5/19)	53.17 ± 10.11/52.87 ± 10.07	29.76 ± 11.49 mo/30.07 ± 11.10 mo	DJD + C	MTX + SSZ + Diclofenac	24 wk	5.6.7.16.17.18.
Zhao et al (2021b)	24(6/18)/24(5/19)	53.17 ± 10.11/52.87 ± 10.07	29.76 ± 11.49 mo/30.07 ± 11.10 mo	DJD + C	MTX + SSZ + Diclofenac	24 wk	2.3.12.13.14.15.
Zheng (2018)	30(7/23)/30(5/25)	50.67 ± 11.58/49.63 ± 12.82	41.23 ± 7.58 mo/38.50 ± 7.29 mo	DJD + C	MTX + Folic Acid + Ferrous Sulfate	8 wk	1.2.3.4.12.13.14.19.

Values are mean ± SD.

1. effective rate; 2. ESR; 3. CRP; 4. RF; 5. TNF-α; 6. IL-6; 7. IL-1β; 8. IL-1; 9. CD4^+^; 10. CD8^+^; 11. CD4^+^/CD8^+^; 12. duration of morning stiffness; 13. number of joint tenderness; 14. number of swollen joints; 15. grip strength of both hands; 16. VAS; 17. HAQ; 18. DAS-28; 19. AEs.

C = control group, DJD = Duhuo-Jisheng decoction, HCQ = Hydroxychloroquine sulfate, LEF = Leflunomide, MTX = methotrexate, SSZ = sulfasalazine, T = experimental group.

### 3.2. Bias assessment

**Figure 2.**
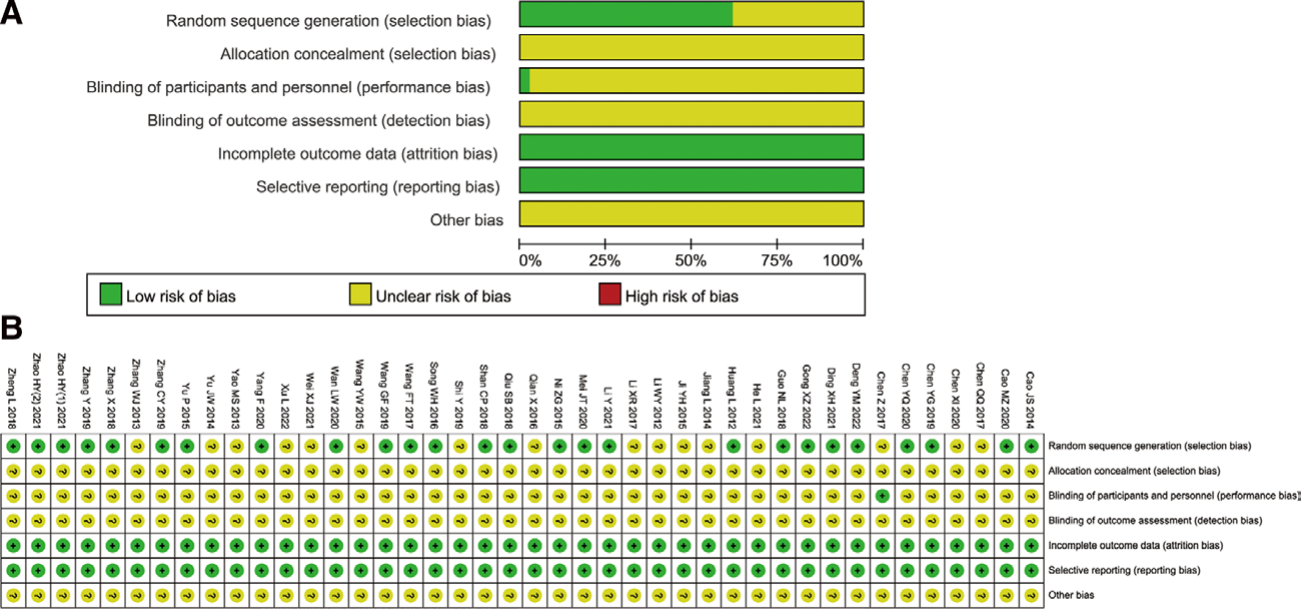
Risk of bias assessments of included studies. (A) overall risk; (B) detailed risk.

All studies mentioned randomization and 26 studies described the method of randomization as a random number table.^[11,13–17,24,25,27–29,35,36,38,39,41–44,49,50,52–56]^ Therefore, in terms of selection bias, the 26 studies were rated as low risk, and the rest were rated as unclear risk. Only one research performed blinding of participants and personnel (Fig. 2).^[26]^ Overall, the quality of included studies is not high.

### 3.3. Effective rate

**Figure 3.**
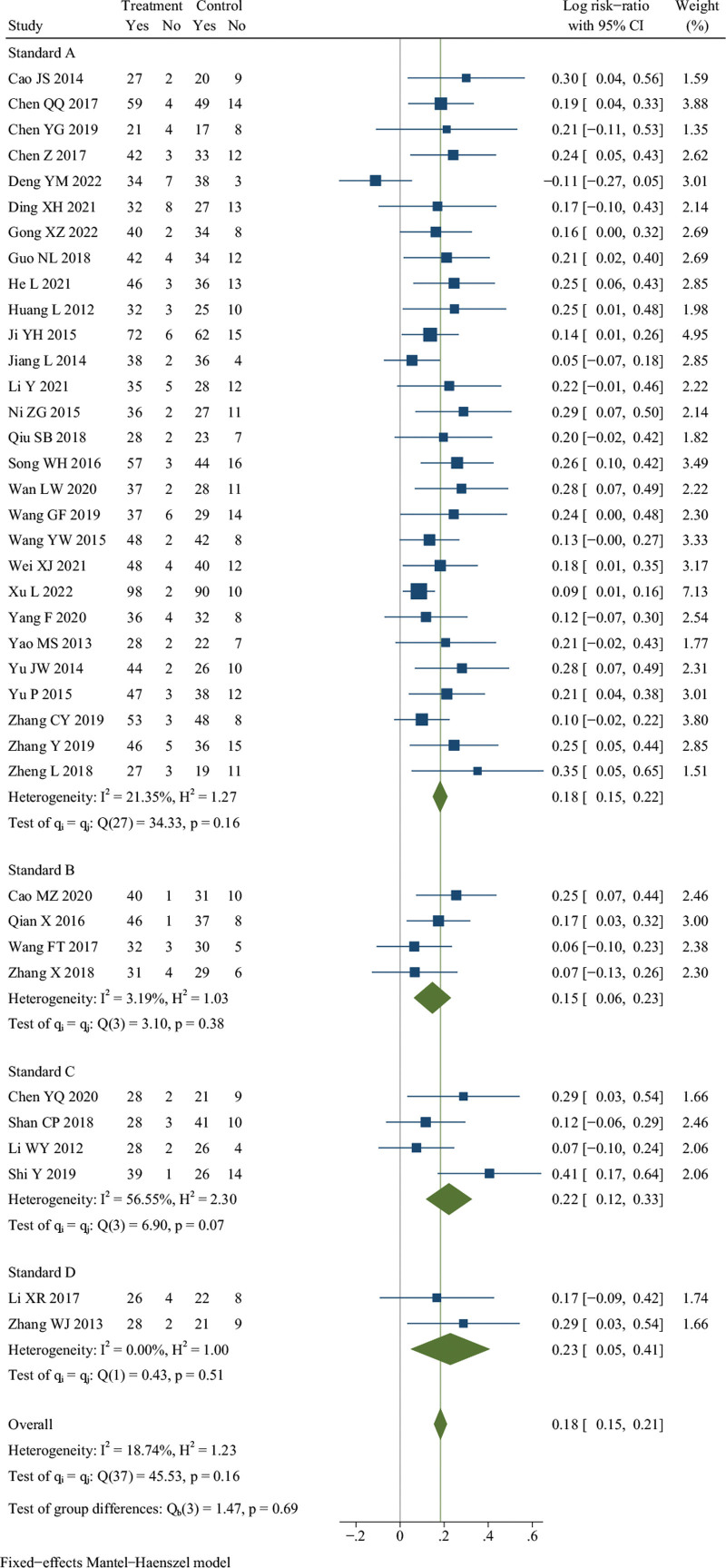
Forest plot of effective rate. Standard A: Guiding Principles for Clinical Research of New Traditional Chinese Medicine; Standard B: ACR20, ACR50, ACR70; Standard C: Guidelines for the diagnosis and treatment of rheumatoid arthritis (Rheumatology Branch of the Chinese Medical Association); Standard D: Traditional Chinese and Western Medicine Diagnosis and Treatment of Rheumatology.

Thirty-eight studies reported the effective rate.^[11–15,17,18,24–53,56]^ There were 1692 patients in the treatment group and 1679 in the control group. Meta-analysis indicated that there was no significant difference in the effective rate of DJD treatment compared to rDMARDs (*I*^2^ = 18.74%, fixed effects model, RR = 0.18, 95% CI [0.15, 0.21], *P* = .16, Fig. 3).

However, the results of subgroup analysis showed that, only when the course of treatment was greater than or equal to 12 weeks, the effective rate of DJD treatment was significantly higher than rDMARDs (*I*^2^ = 44.93%, fixed effects model, RR = 0.20, 95% CI [0.15, 0.24], *P* = .03) (see Table S2, Supplemental Digital Content, http://links.lww.com/MD/K289, Figure S1C, Supplemental Digital Content, http://links.lww.com/MD/K260).

### 3.4. Erythrocyte sedimentation rate (ESR)

**Figure 4.**
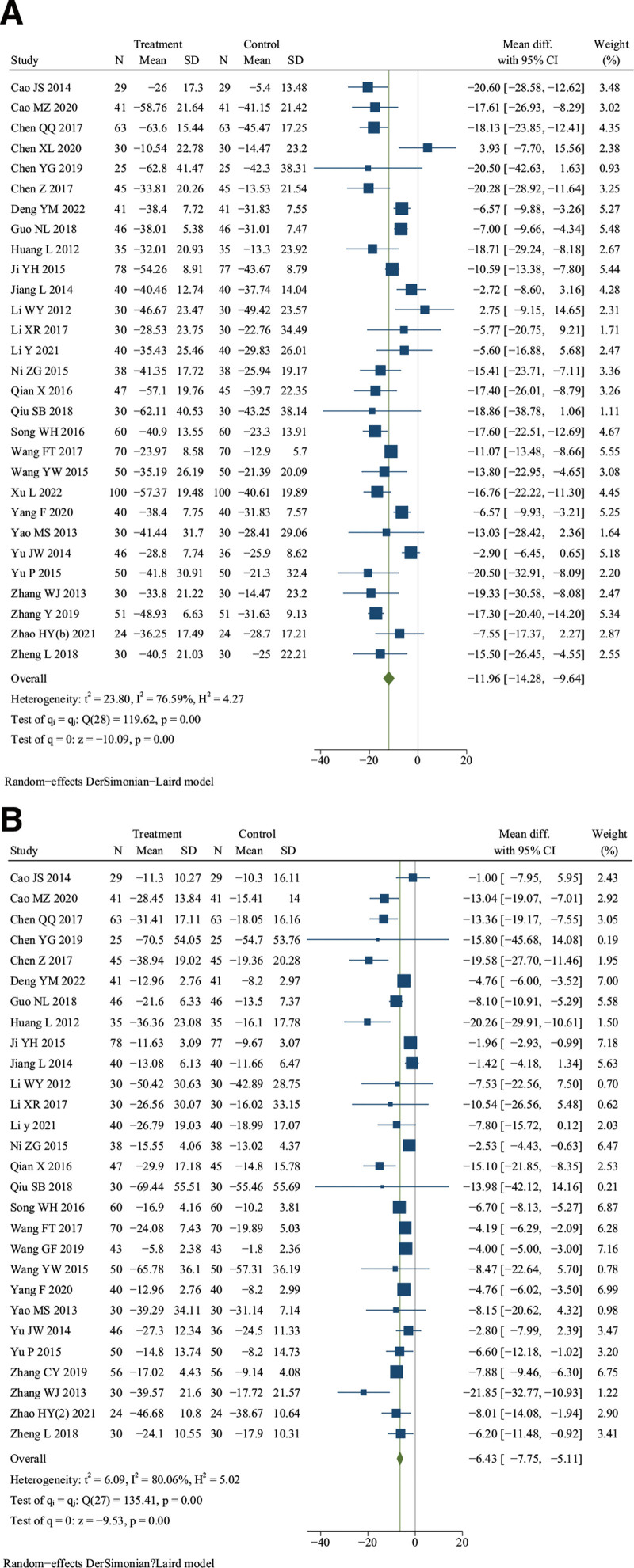
Forest plot of (A) erythrocyte sedimentation rate (ESR) and (B) C-reactive protein (CRP).

Twenty-nine studies reported ESR^.[11–15,17,18,23,24,26,27,31–38,41,43,45,47–49,51,53,55–72]^ There were 1269 patients in the treatment group and 1256 in the control group. Meta-analysis indicated that DJD therapy had a better effect in lowering the level of ESR compared with rDMARDs (*I*^2^ = 76.59%, random effects model, WMD = −11.96, 95% CI [−14.28, −9.64], *P* < .001, Fig. 4A).

The results of all subgroup analysis were consistent with the overall research results (see Table S2, Supplemental Digital Content, http://links.lww.com/MD/K289, Figure S2, Supplemental Digital Content, http://links.lww.com/MD/K261). And there was a significant heterogeneity difference between different subgroups of the control group only (*P* = .04) (see Table S2, Supplemental Digital Content, http://links.lww.com/MD/K289, Figure S2, Supplemental Digital Content, http://links.lww.com/MD/K261). The meta-regression results also showed that the different intervention measures of the control group might be a reason for heterogeneity (*p* =. 02) (see Table S2, Supplemental Digital Content, http://links.lww.com/MD/K289). In addition, sensitivity analysis revealed that the significant heterogeneity might be caused by 4 studies,^[14,26,37,51]^ and it was reduced after these studies were excluded (*I*^2^ = 49.82%, fixed effects model, WMD = −11.67, 95% CI [−12.68, −10.66], *P* < .001) (see Figure S3, Supplemental Digital Content, http://links.lww.com/MD/K262). Together, these results provide important insights that the level of ESR could be better reduced by DJD than rDMARDs.

### 3.5. C-reactive protein (CRP)

Twenty-eight studies reported CRP^.[11–15,17,18,24,26,27,31–38,41,43,45,47–49,51,53,55–57,59–62,65–67,69,70,72]^ There were 1187 patients in the treatment group and 1174 in the control group. Meta-analysis showed that DJD therapy could significantly reduce the level of CRP than rDMARDs (*I*^2^ = 80.06%, random effects model, WMD = −6.43, 95% CI [−7.75, −5.11], *P* < .001, Fig. 4B).

Except for the meta-analysis result of the control group using multiple drugs (*P* = .63), the results of other subgroup analysis were consistent with the overall research results (see Table S2, Supplemental Digital Content, http://links.lww.com/MD/K289, Figure S4, Supplemental Digital Content, http://links.lww.com/MD/K263). And there were significant heterogeneity differences between different subgroups of the experimental group (*P* < .001) and the control group (*P* < .001) (see Table S2, Supplemental Digital Content, http://links.lww.com/MD/K289, Figure S4, Supplemental Digital Content, http://links.lww.com/MD/K263). The meta-regression results also showed that the different intervention measures of the experimental group (*P* = .004) and the control group (*P* = .04) might cause heterogeneity (see Table S2, Supplemental Digital Content, http://links.lww.com/MD/K289). Furthermore, sensitivity analysis indicated that the significant heterogeneity might be caused by 4 studies,^[14,26,37,51]^ and it was decreased after these studies were excluded (*I*^2^ = 45.25%, fixed effects model, WMD = −4.45, 95% CI [−4.89, −4.02], *P* < .001) (see Figure S5, Supplemental Digital Content, http://links.lww.com/MD/K264). Overall, these results provide significant evidence that the level of CRP could be better lowered by DJD than rDMARDs.

### 3.6. Rheumatoid factor (RF)

**Figure 5.**
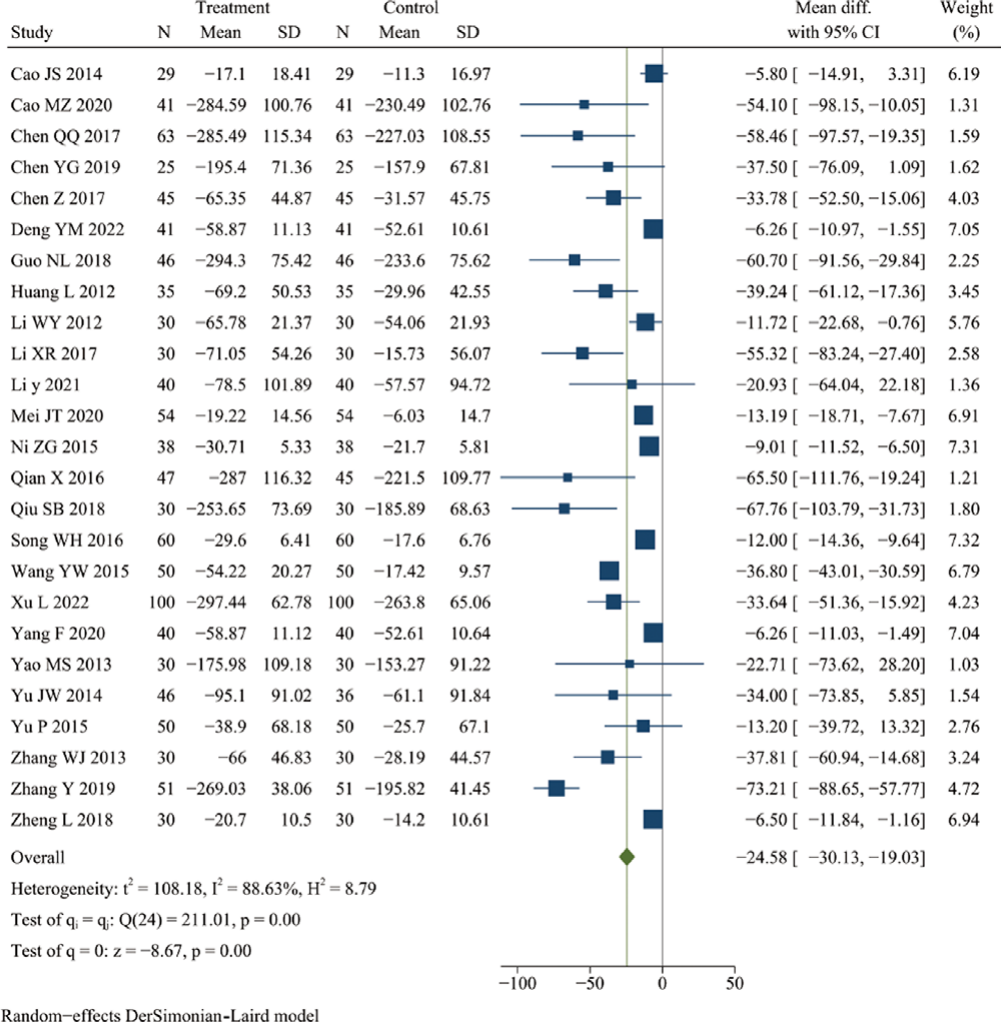
Forest plot of rheumatoid factor (RF).

Twenty-five studies reported RF.^[11–18,24,26,27,33–38,41,45,47–49,51,53,56]^ There were 1081 patients in the treatment group and 1069 in the control group. Meta-analysis revealed that DJD treatment had a better effect in reducing the level of RF than rDMARDs (*I*^2^ = 88.63%, random effects model, WMD = −24.58, 95% CI [−30.13, −10.93], *P* < .001, Fig. 5).

The results of all subgroup analysis were consistent with the overall research results (see Table S2, Supplemental Digital Content, http://links.lww.com/MD/K289, Figure S6, Supplemental Digital Content, http://links.lww.com/MD/K265). And there were significant heterogeneity differences between different subgroups of course of disease (*P* < .001), course of treatment (*P* = .01), the experimental group (*P* < .001), the control group (*P* = .02), and random sequence generation (*P* < .001) (see Table S2, Supplemental Digital Content, http://links.lww.com/MD/K289, Figure S6, Supplemental Digital Content, http://links.lww.com/MD/K265). However, the meta-regression failed to identify the possible cause of heterogeneity (see Table S2, Supplemental Digital Content, http://links.lww.com/MD/K289). Additionally, sensitivity analysis showed that the significant heterogeneity might be caused by 5 studies,^[13,34,38,45,53]^ and it was decreased after these studies were excluded (*I*^2^ = 46.26%, fixed effects model, WMD = −0.31, 95% CI [−10.38, −9.01], *P* < .001) (see Figure S7, Supplemental Digital Content, http://links.lww.com/MD/K266). Taken together, these results suggest that the level of RF could be better reduced by DJD than rDMARDs.

### 3.7. Tumor necrosis factor-α (TNF-α)

**Figure 6.**
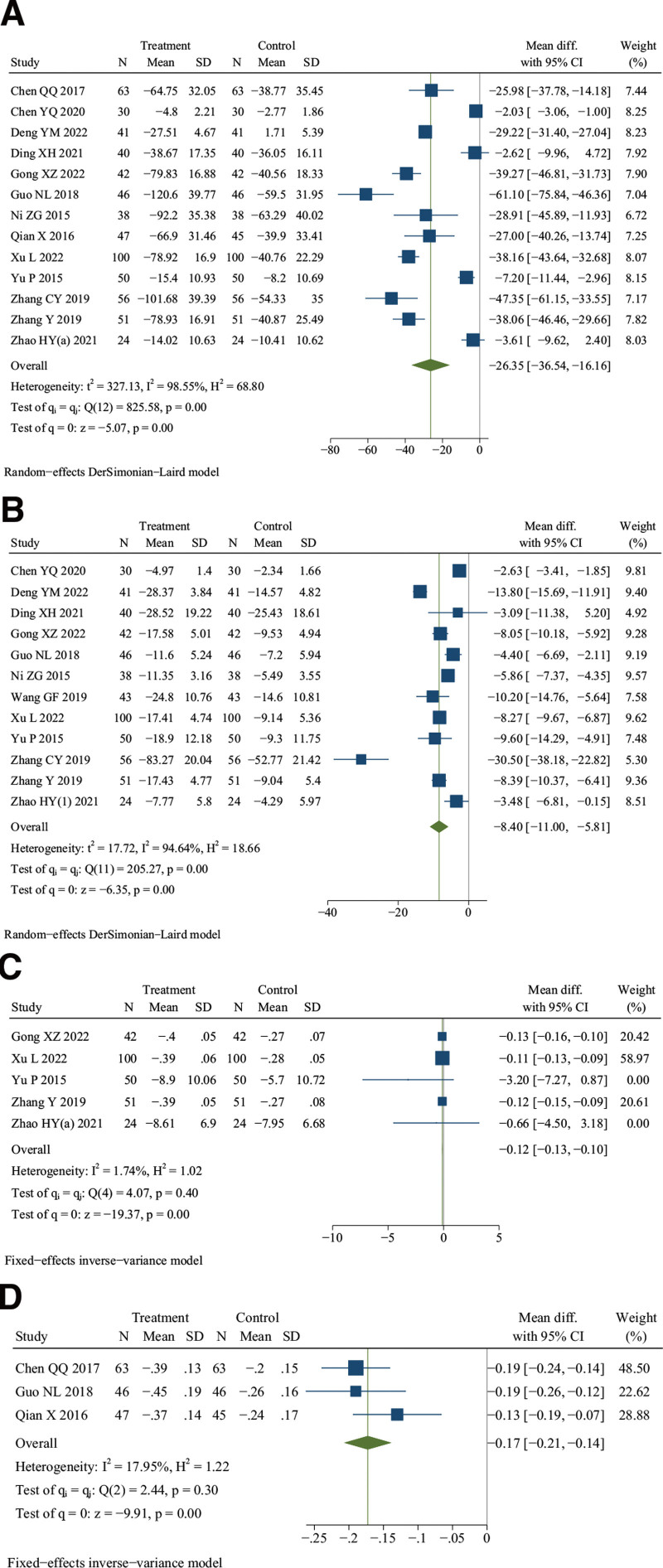
Forest plot of (A) tumor necrosis factor-α (TNF-α), (B) interleukin 6 (IL-6), (C) interleukin 1β (IL-1β), and (D) interleukin 1 (IL-1).

Thirteen studies reported TNF-α.^[12,13,25,27–29,36,37,47,49,50,53,54]^ There were 628 patients in the treatment group and 626 in the control group. Meta-analysis showed that DJD treatment could significantly lower the level of TNF-α than rDMARDs (*I*^2^ = 98.55%, random effects model, WMD = −26.35, 95% CI [−36.54, −16.16], *P* < .001, Fig. 6A).

Except for the meta-analysis result of the subgroup of unclear risk (*P* = .09), the results of other subgroup analysis were consistent with the overall research results (see Table S2, http://links.lww.com/MD/K289, Figure S8, Supplemental Digital Content, http://links.lww.com/MD/K267). And there were significant heterogeneity differences between different subgroups of age (*P* = .03) and the experimental group (*P* < .001) (see Table S2, Supplemental Digital Content, http://links.lww.com/MD/K289, Figure S8, Supplemental Digital Content, http://links.lww.com/MD/K267). However, the meta-regression results failed to identify the possible cause of heterogeneity (see Table S2, Supplemental Digital Content, http://links.lww.com/MD/K289). In addition, sensitivity analysis showed that the significant heterogeneity might be caused by 2 studies,^[13,50]^ and it was decreased after these studies were excluded (*I*^2^ = 51.56%, random effects model, WMD = −21.72, 95% CI [−32.34, −11.10], *P* < .001) (see Figure S9, Supplemental Digital Content, http://links.lww.com/MD/K268). In summary, these results indicate that the level of TNF-α could be better lowered by DJD than rDMARDs.

### 3.8. Interleukin 6 (IL-6)

Twelve studies reported IL-6.^[13,25,27–29,36,44,47,49,50,53,54]^ There were 561 patients in the treatment group and 561 in the control group. Meta-analysis indicated that DJD treatment could significantly reduce the level of IL-6 than rDMARDs (*I*^2^ = 94.64%, random effects model, WMD = −8.40, 95% CI [−11.00, −5.81], *P* < .001, Fig. 6B).

Except for the meta-analysis result of the experimental group using DJD only (*P* = .14), the results of other subgroup analysis were consistent with the overall research results (see Table S2, Supplemental Digital Content, http://links.lww.com/MD/K289, Figure S10, Supplemental Digital Content, http://links.lww.com/MD/K268). And there was no significant heterogeneity difference between different subgroups (see Table S2, Supplemental Digital Content, http://links.lww.com/MD/K289, Figure S10, Supplemental Digital Content, http://links.lww.com/MD/K268). The meta-regression results showed that the different age (*P* = .003), random sequence generation (*P* = .03), and the different intervention measures of the control group might cause heterogeneity (*P* = .02) (see Table S2, Supplemental Digital Content, http://links.lww.com/MD/K289). Furthermore, sensitivity analysis indicated that the significant heterogeneity might be caused by 2 studies,^[27,50]^ and it was decreased after these studies were excluded (*I*^2^ = 49.65%, fixed effects model, WMD = −6.40, 95% CI [−8.41, −4.39], *P* < .001) (see Figure S11, Supplemental Digital Content, http://links.lww.com/MD/K270). Overall, these results show that DJD treatment could better lower the level of IL-6 than rDMARDs.

### 3.9. Interleukin 1β (IL-1β)

Five studies reported IL-1β.^[29,47,49,53,54]^ There were 267 patients in the treatment group and 267 in the control group. Meta-analysis indicated that DJD treatment could significantly reduce the level of IL-1β than rDMARDs (*I*^2^ = 1.74%, fixed effects model, WMD = −0.12, 95% CI [−0.13, −0.10], *P* < .001, Fig. 6C). Overall, these results show that DJD treatment could better lower the level of IL-1β than rDMARDs.

### 3.10. Interleukin 1 (IL-1)

Three studies reported IL-1.^[12,13,37]^ There were 156 patients in the treatment group and 154 in the control group. Meta-analysis indicated that DJD treatment could significantly reduce the level of IL-1 than rDMARDs (*I*^2^ = 17.95%, fixed effects model, WMD = −0.17, 95% CI [−0.21, −0.14], *P* < .001, Fig. 6D). Overall, these results show that DJD treatment could better lower the level of IL-1 than rDMARDs.

### 3.11. T lymphocyte subpopulation

**Figure 7.**
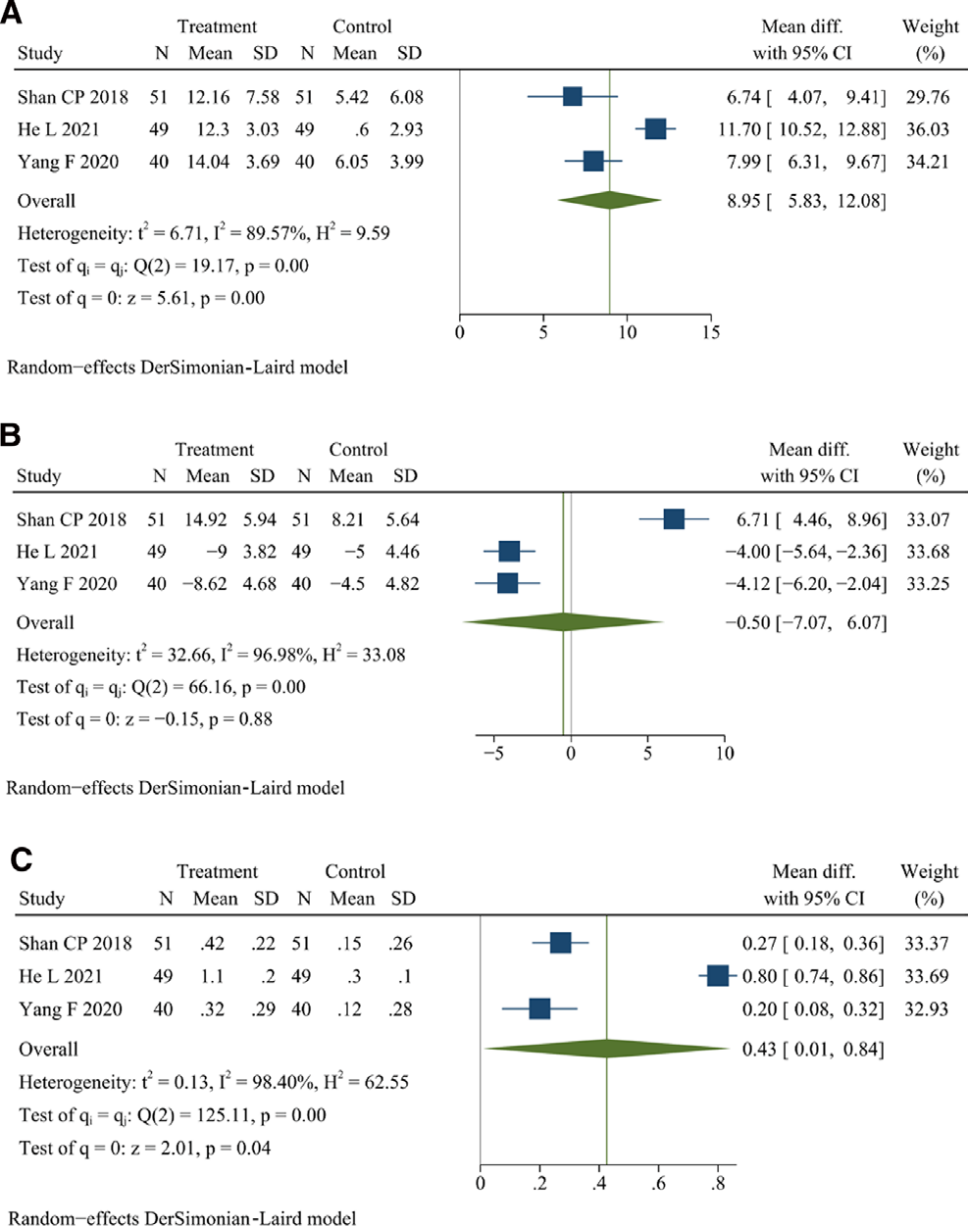
Forest plot of (A) CD4^+^, (B) CD8^+^, and (C) CD4^+^/CD8^+^.

Three studies reported CD4^+^, CD8^+^, and CD4^+^/CD8^+^.^[17,30,39]^ There were 140 patients in the treatment group and 140 in the control group. Meta-analysis revealed that DJD treatment had a significant effect in improving the level of CD4^+^ (*I*^2^ = 89.57%, random effects model, WMD = 8.95, 95% CI [5.83, 12.08], *P* < .001, Fig. 7A), CD8^+^ (*I*^2^ = 96.98%, random effects model, WMD = −0.50, 95% CI [−7.07, −6.07], *P* = .88, Fig. 7B), and CD4^+^/CD8^+^ (*I*^2^ = 98.40%, random effects model, WMD = 0.43, 95% CI [0.01, 0.84], *P* = .04, Fig. 7C) than rDMARDs. Sensitivity analysis failed to identify the studies with possible heterogeneity in CD4^+^, CD8^+^, and CD4^+^/CD8^+^, as only 3 studies were included (see Figure S12, Supplemental Digital Content, http://links.lww.com/MD/K271). The results in this section illustrate that the level of CD4^+^, CD8^+^, and CD4^+^/CD8^+^ could be better improved by DJD than by rDMARDs.

### 3.12. Duration of morning stiffness

**Figure 8.**
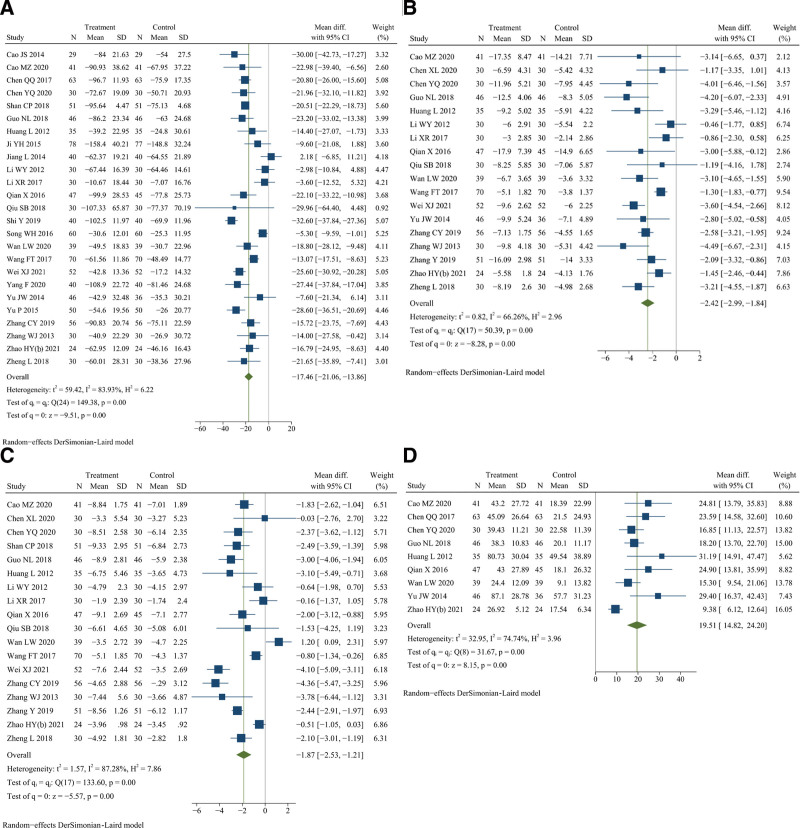
Forest plot of (A) duration of morning stiffness, (B) number of joint tenderness, (C) number of swollen joints, and (D) grip strength of both hands.

Twenty-five studies reported duration of morning stiffness.^[11–15,17,18,25,31–34,37–43,46,49–51,55,56]^ There were 1087 patients in the treatment group and 1074 in the control group. Meta-analysis indicated that DJD treatment had a better effect on reducing the duration of morning stiffness than rDMARDs (*I*^2^ = 83.93%, random effects model, WMD = −17.46, 95% CI [−21.06, −13.86], *P* < .001, Fig. 8A).

Except for the meta-analysis result of the experimental group using only DJD (*P* = .07), the results of other subgroup analysis were consistent with the overall research results (see Table S2, Supplemental Digital Content, http://links.lww.com/MD/K289, Figure S13, Supplemental Digital Content, http://links.lww.com/MD/K272). And there was no significant heterogeneity difference between different subgroups (see Table S2, http://links.lww.com/MD/K289, Figure S13, Supplemental Digital Content, http://links.lww.com/MD/K272). The meta-regression failed to identify the possible cause of heterogeneity (see Table S2, Supplemental Digital Content, http://links.lww.com/MD/K289). In addition, sensitivity analysis showed that the significant heterogeneity might be caused by 5 studies,^[32–34,40,41]^ and it was decreased after these studies were excluded (*I*^2^ = 43.45%, fixed effects model, WMD = −20.11, 95% CI [−21.42, −18.79], *P* < .001) (see Figure S14, Supplemental Digital Content, http://links.lww.com/MD/K273). Overall, these results show that the duration of morning stiffness could be better reduced by DJD than rDMARDs.

### 3.13. Number of joint tenderness

Eighteen studies reported the number of joint tenderness.^[11,13,14,18,23,25,33,34,37,38,42,43,46,50,51,53,55,56]^ There were 717 patients in the treatment group and 705 in the control group. Meta-analysis indicated that DJD treatment had a better effect on reducing the number of joint tenderness than rDMARDs (*I*^2^ = 66.26%, random effects model, WMD = −2.42, 95% CI [−2.99, −1.84], *P* < .001, Fig. 8B).

Except for the meta-analysis results of the course of treatment greater than or equal to 12 weeks (*P* = .06) and the experimental group used DJD only (*P* = .16), the results of other subgroup analysis were consistent with the overall research results (see Table S2, Figure S15, Supplemental Digital Content, http://links.lww.com/MD/K274). And there was no significant heterogeneity difference between different subgroups (see Table S2, http://links.lww.com/MD/K289, Figure S15, Supplemental Digital Content, http://links.lww.com/MD/K274). The meta-regression failed to identify the possible cause of heterogeneity (see Table S2, Supplemental Digital Content, http://links.lww.com/MD/K289). Furthermore, sensitivity analysis showed that the significant heterogeneity might be caused by 2 studies,^[33,46]^ and it was decreased after these studies were excluded (*I*^2^ = 56.70%, random effects model, WMD = −2.42, 95% CI [−2.98, −1.86], *P* < .001) (see Figure S16, Supplemental Digital Content, http://links.lww.com/MD/K275). Overall, these results show that the number of joint tenderness could be better reduced by DJD than rDMARDs.

### 3.14. Number of swollen joints

Eighteen studies reported the number of swollen joints.^[11,13,14,23,25,33,34,37–39,42,43,46,50,51,53,55,56]^ There were 722 patients in the treatment group and 720 in the control group. Meta-analysis indicated that DJD treatment had a better effect on reducing the number of swollen joints than rDMARDs (*I*^2^ = 87.28%, random effects model, WMD = −1.87, 95% CI [−2.53, −1.21], *P* < .001, Fig. 8C).

Except for the meta-analysis result of the experimental group using DJD only (*P* = .41), the results of other subgroup analysis were consistent with the overall research results (see Table S2, Supplemental Digital Content, http://links.lww.com/MD/K289, Figure S17, Supplemental Digital Content, http://links.lww.com/MD/K276). And there was a significant heterogeneity difference only between different subgroups of the experimental group (*P* < .001) (see Table S2, Supplemental Digital Content, http://links.lww.com/MD/K289, Figure S17, Supplemental Digital Content, http://links.lww.com/MD/K276). The meta-regression failed to identify the possible cause of heterogeneity (see Table S2, Supplemental Digital Content, http://links.lww.com/MD/K289). Additionally, sensitivity analysis showed that the significant heterogeneity might be caused by 3 studies,^[42,46,50]^ and it was decreased after these studies were excluded (*I*^2^ = 50.11%, random effects model, WMD = −1.72, 95% CI [−2.27, −1.17], *P* < .001) (see Figure S18, Supplemental Digital Content, http://links.lww.com/MD/K277). Overall, these results show that the number of swollen joints could be better reduced by DJD than rDMARDs.

### 3.15. Grip strength of both hands

Nine studies reported the grip strength of both hands.^[11–14,18,25,37,42,55]^ There were 371 patients in the treatment group and 359 in the control group. Meta-analysis indicated that DJD treatment had a better effect on improving the grip strength of both hands than rDMARDs (*I*^2^ = 74.74%, random effects model, WMD = 19.51, 95% CI [14.82, 24.20], *P* < .001, Fig. 8D).

Except for the meta-analysis results of age less than 47 years old (*P* = .33), the course of disease greater than or equal to 4 years (*P* = .14), the course of treatment less than 12 weeks (*P* = .20), the control group used single drug (*P* = .37), and random sequence generation with unclear risk (*P* = .77), the results of other subgroup analysis were consistent with the overall research results (see Table S2, Supplemental Digital Content, http://links.lww.com/MD/K289, Figure S19, Supplemental Digital Content, http://links.lww.com/MD/K278). And there were significant heterogeneity differences between different subgroups of age (*P* = .01) and the random sequence generation (*P* = .04) (see Table S2, Supplemental Digital Content, http://links.lww.com/MD/K289, Figure S19, Supplemental Digital Content, http://links.lww.com/MD/K278). The meta-regression failed to identify the possible cause of heterogeneity (see Table S2, Supplemental Digital Content, http://links.lww.com/MD/K289). In addition, sensitivity analysis showed that the significant heterogeneity might be caused by one study,^[55]^ and it was decreased after this study was excluded (*I*^2^ = 29.67%, fixed effects model, WMD = 19.29, 95% CI [16.69, 21.88], *P* < .001) (see Figure S20, Supplemental Digital Content, http://links.lww.com/MD/K279). Overall, these results show that the grip strength of both hands could be better improved by DJD than rDMARDs.

### 3.16. Visual analogue scale (VAS)

**Figure 9.**
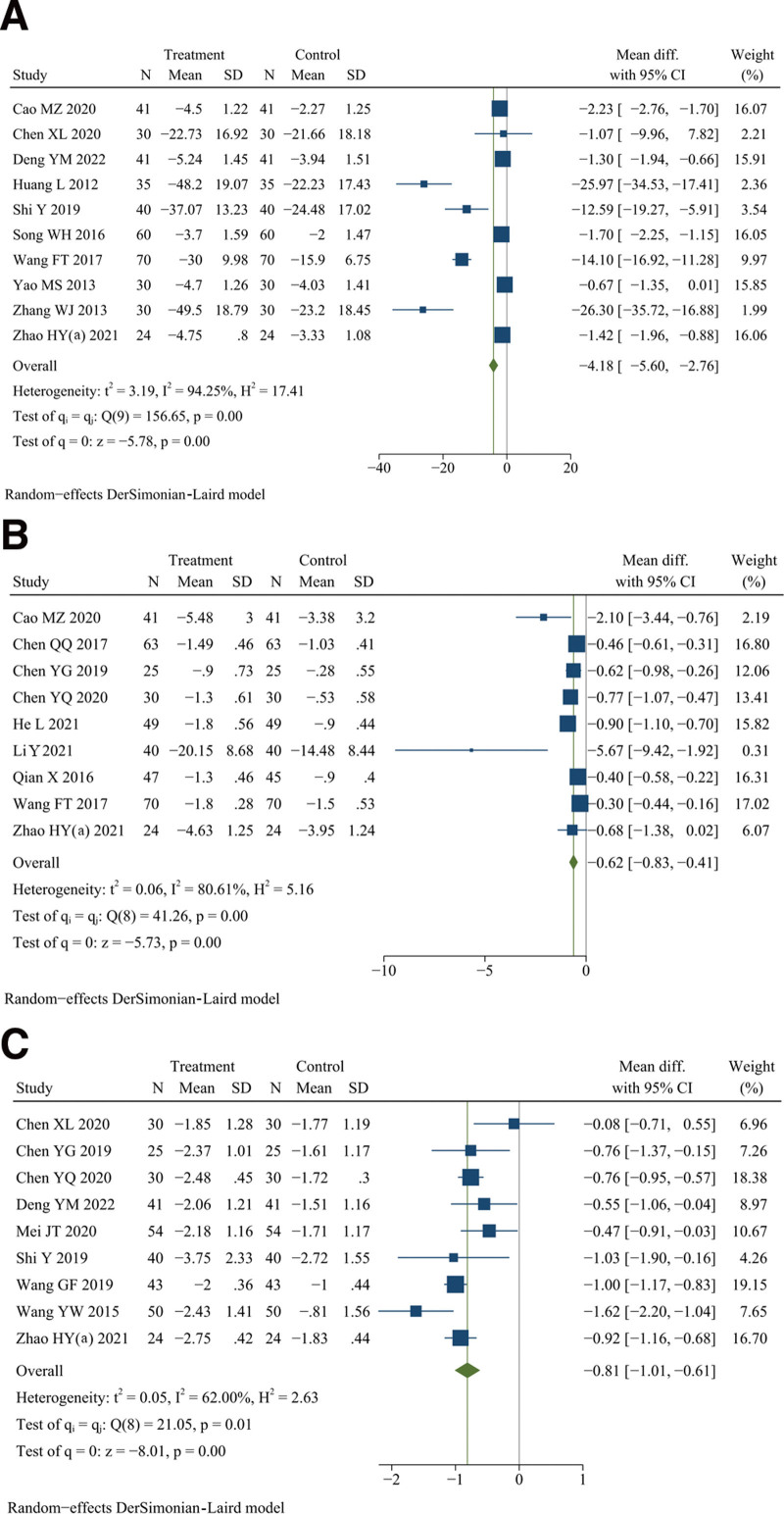
Forest plot of (A) visual analogue scale (VAS), (B) health assessment questionnaire (HAQ), and (C) disease activity score in 28 joints (DAS-28).

Ten studies reported the score of VAS.^[11,14,23,27,40,41,43,48,51,54]^ There were 401 patients in the treatment group and 401 in the control group. Meta-analysis indicated that DJD treatment had a better effect on reducing the score of VAS than rDMARDs (*I*^2^ = 94.25%, random effects model, WMD = −4.18, 95% CI [−5.60, −2.76], *P* < .001, Fig. 9A).

Except for the meta-analysis result of age greater than or equal to 47 years old (*P* = .47), the results of other subgroup analysis were consistent with the overall research results (see Table S2, Supplemental Digital Content, http://links.lww.com/MD/K289, Figure S21, Supplemental Digital Content, http://links.lww.com/MD/K280). And there was a significant heterogeneity difference only between different subgroups of age (*P* < .001) (see Table S2, Supplemental Digital Content, http://links.lww.com/MD/K289, Figure S21, Supplemental Digital Content, http://links.lww.com/MD/K280). The meta-regression failed to identify the possible cause of heterogeneity (see Table S2, Supplemental Digital Content, http://links.lww.com/MD/K289). Furthermore, sensitivity analysis showed that the significant heterogeneity might be caused by 5 studies,^[11,14,40,43,51]^ and it was decreased after these studies were excluded (*I*^2^ = 27.61%, fixed effects model, WMD = −1.33, 95% CI [−1.63, −1.04], *P* < .001) (see Figure S22, Supplemental Digital Content, http://links.lww.com/MD/K281). Overall, these results show that the score of VAS could be better reduced by DJD than rDMARDs.

### 3.17. Health assessment questionnaire (HAQ)

Nine studies reported the score of HAQ.^[11,12,24,25,30,35,37,43,54]^ There were 389 patients in the treatment group and 387 in the control group. Meta-analysis indicated that DJD treatment had a better effect on reducing the score of HAQ than rDMARDs (*I*^2^ = 80.61%, random effects model, WMD = −0.62, 95% CI [−0.83, −0.41], *P* < .001, Fig. 9B).

Except for the meta-analysis results of age greater than or equal to 47 years old (*P* = .08), the course of disease less than 4 years (*P* = .15), the course of treatment greater than or equal to 12 weeks (*P* = .18), and the control group used single drug (*P* = .05), the results of other subgroup analysis were consistent with the overall research results (see Table S2, Supplemental Digital Content, http://links.lww.com/MD/K289, Figure S23, Supplemental Digital Content, http://links.lww.com/MD/K282). And there was a significant heterogeneity difference only between different subgroups of age (*P* = .02) (see Table S2, Supplemental Digital Content, http://links.lww.com/MD/K289, Figure S23, Supplemental Digital Content, http://links.lww.com/MD/K282). The meta-regression failed to identify the possible cause of heterogeneity (see Table S2, Supplemental Digital Content, http://links.lww.com/MD/K289). Additionally, sensitivity analysis showed that the significant heterogeneity might be caused by 3 studies,^[11,30,35]^ and it was decreased after these studies were excluded (*I*^2^ = 50.02%, random effects model, WMD = −0.47, 95% CI [−0.60, −0.33], *P* < .001) (see Figure S24, Supplemental Digital Content, http://links.lww.com/MD/K283). Overall, these results show that the score of HAQ could be better reduced by DJD than rDMARDs.

### 3.18. Disease activity score in 28 joints (DAS-28)

Nine studies reported the score of DAS-28.^[16,23–25,27,40,44,45,54]^ There were 337 patients in the treatment group and 337 in the control group. Meta-analysis indicated that DJD treatment had a better effect on lowering the score of DAS-28 than rDMARDs (*I*^2^ = 62.00%, random effects model, WMD = −0.81, 95% CI [−1.01, −0.61], *P* < .001, Fig. 9C).

Except for the meta-analysis results of age greater than or equal to 47 years old (*P* = .31), the course of disease less than 4 years (*P* = .05), the experimental group used DJD plus rDMARDs (*P* = .06), the control group used multiple drugs (*P* = .09), and random sequence generation with low risk (*P* = .14), the results of other subgroup analysis were consistent with the overall research results (see Table S2, Supplemental Digital Content, http://links.lww.com/MD/K289, Figure S25, Supplemental Digital Content, http://links.lww.com/MD/K284). And there was no significant heterogeneity difference between different subgroups (see Table S2, Supplemental Digital Content, http://links.lww.com/MD/K289, Figure S25, Supplemental Digital Content, http://links.lww.com/MD/K284). The meta-regression failed to identify the possible cause of heterogeneity (see Table S2, Supplemental Digital Content, http://links.lww.com/MD/K289). In addition, sensitivity analysis showed that the significant heterogeneity might be caused by 2 studies,^[44,45]^ and it was decreased after these studies were excluded (*I*^2^ = 32.04%, fixed effects model, WMD = −0.74, 95% CI [−0.87, −0.61], *P* < .001) (see Figure S26, Supplemental Digital Content, http://links.lww.com/MD/K285). Overall, these results show that the score of DAS-28 could be better lowered by DJD than rDMARDs.

### 3.19. Adverse events (AEs)

**Figure 10.**
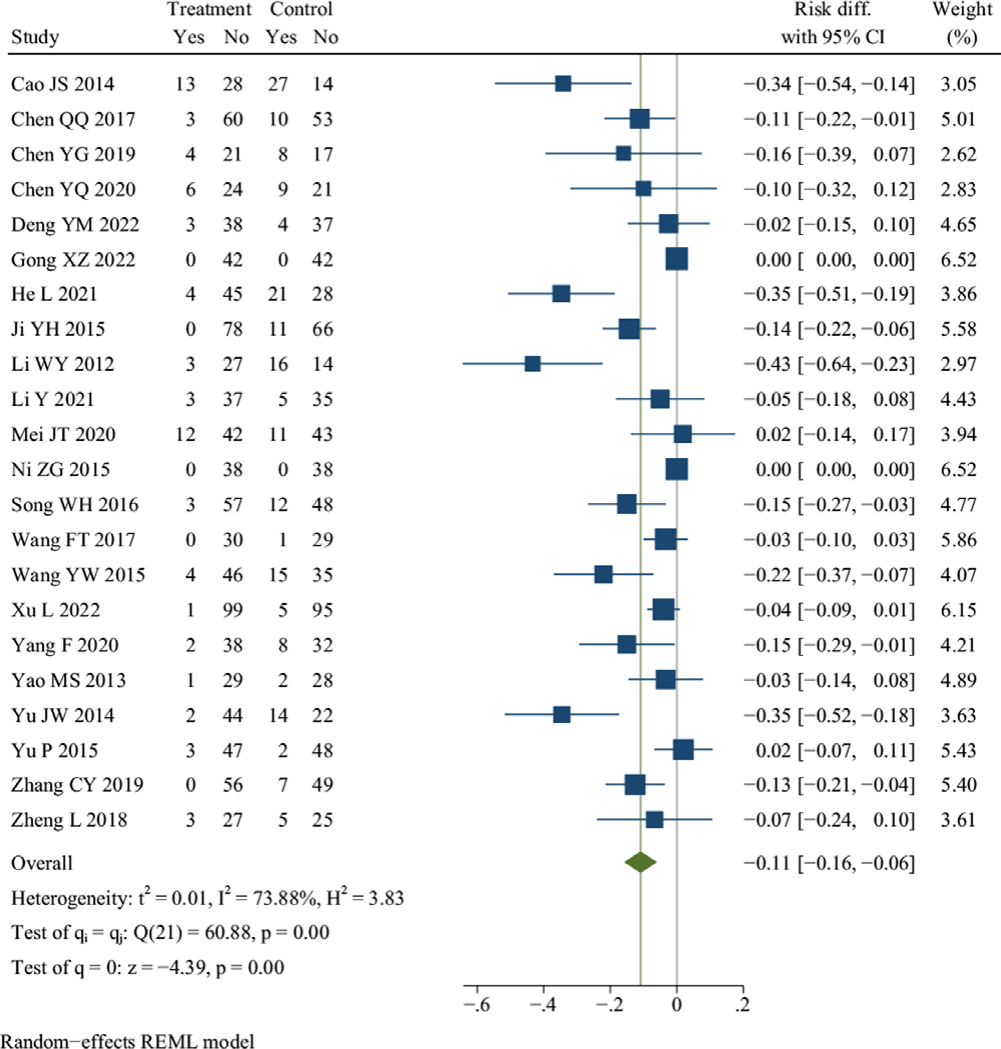
Forest plot of adverse events (AEs).

Twenty-two studies reported AEs.^[12,15–18,24,25,27,29–31,33,35,36,41,43,45,47–50,56]^ There were 1051 patients in the treatment group and 1040 in the control group. The AEs included abnormal liver function, decreased white blood cells, rash, hair loss, dizziness, nausea, vomiting, bloating, diarrhea, etc. These symptoms were mild, tolerable, and could be relieved automatically. Meta-analysis revealed that there was a significant difference in terms of adverse events between DJD treatment and rDMARDs (*I*^2^ = 73.88%, random effects model, RD = −0.11, 95% CI [−0.16, −0.06], *P* < .001, Fig. 10).

Except for the meta-analysis results of the experimental group only used DJD (*P* = .07) and random sequence generation with low risk (*P* = .11), the results of other subgroup analysis were consistent with the overall research results (see Table S2, http://links.lww.com/MD/K289, Figure S27, Supplemental Digital Content, http://links.lww.com/MD/K286). And there was a significant heterogeneity difference only between different subgroups of random sequence generation (*P* = .01) (see Table S2, Supplemental Digital Content, http://links.lww.com/MD/K289, Figure S27, Supplemental Digital Content). The meta-regression failed to identify the possible cause of heterogeneity (see Table S2, Supplemental Digital Content, http://links.lww.com/MD/K289). Additionally, sensitivity analysis showed that the significant heterogeneity might be caused by 3 studies,^[18,30,33]^ and it was decreased after these studies were excluded (*I*^2^ = 38.84%, fixed effects model, RD = −0.07, 95% CI [−0.10, −0.05], *P* < .001) (see Figure S28, Supplemental Digital Content, http://links.lww.com/MD/K287). Taken together, these results indicate that DJD treatment may be safer than rDMARDs.

### 3.20. Publication bias

**Figure 11.**
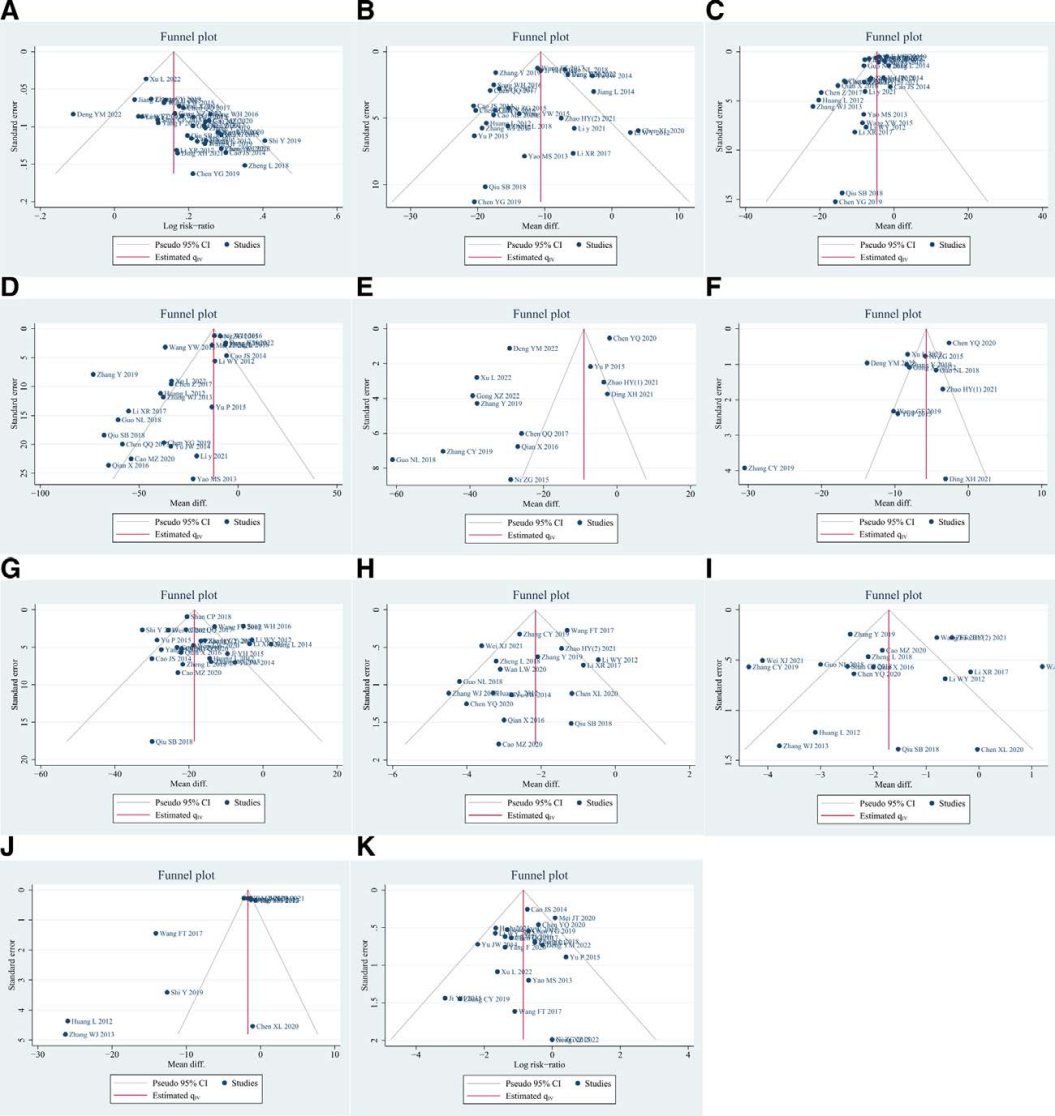
Funnel plot of publication bias. (A) Effective rate (Egger’s test: *P* < .001); (B) ESR (Egger’s test: *P* = .351); (C) CRP (Egger’s test: *P* < .001); (D) RF (Egger’s test: *P* < .001); (E) TNF-α (Egger’s test: *P* = .059); (F) IL-6 (Egger’s test: *P* = .105); (G) duration of morning stiffness (Egger’s test: *P* = .729); (H) number of joint tenderness (Egger’s test: *P* = .242); (I) number of swollen joints (Egger’s test: *P* = .754); (J) VAS (Egger’s test: *P* = .008); (K) AEs (Egger’s test: *P* = .153). AEs = adverse events, CRP = C-reactive protein, ESR = erythrocyte sedimentation rate, IL-6 = interleukin 6, RF = rheumatoid factor, TNF-α = tumor necrosis factor-α, VAS = visual analogue scale.

Publication bias was assessed for 11 outcomes, including effective rate, ESR, CRP, RF, TNF-α, IL-6, duration of morning stiffness, number of joint tenderness, number of swollen joints, VAS, and AEs. The results to emerge from the funnel plot and Egger’s test were that there was a publication bias in the effective rate (Egger’s test: *P* < .001, Fig. 11A), CRP (Egger’s test: *P* < .001, Fig. 11C), RF (Egger’s test: *P* < .001, Fig. 11D), and VAS (Egger’s test: *P* = .008, Fig. 11J). However, 7 aspects did not show publication bias, including ESR (Egger’s test: *P* = .351, Fig. 11B), TNF-α (Egger’s test: *P* = .059, Fig. 11E), IL-6 (Egger’s test: *P* = .105, Fig. 11F), duration of morning stiffness (Egger’s test: *P* = .729, Fig. 11G), number of joint tenderness (Egger’s test: *P* = .242, Fig. 11H), number of swollen joints (Egger’s test: *P* = .754, Fig. 11I), and AEs (Egger’s test: *P* = .153, Fig. 11K). Overall, we hold the review that the results of the effective rate, CRP, RF, and VAS should be treated with caution.

## 4. Discussion

### 4.1. Principal findings

Collectively, DJD therapy could be used as an important complementary or alternative therapy for RA. In this study, we conducted a meta-analysis with 3635 subjects in 42 articles to evaluate the efficacy and safety of DJD for RA. Nineteen outcome indicators were evaluated, including effective rate, ESR, CRP, RF, TNF-α, IL-1, IL-1β, IL-6, T lymphocyte subpopulation (including CD4^+^, CD8^+^, and CD4^+^/CD8^+^), duration of morning stiffness, number of joint tenderness, number of swollen joints, grip strength of both hands, VAS, HAQ, DAS-28, AEs. In addition, subgroup analysis, meta-regression, and sensitivity analysis were performed to explore possible sources of heterogeneity, including age, course of disease, course of treatment, interventions used in the experimental or control group, and random sequence generation. The bias assessment showed that the overall quality was not high, especially in the aspects of random sequence generation, allocation concealment, and blinding of participants and personnel. This may be related to the characteristics of TCM syndrome differentiation and treatment, that is, TCM doctors need to face each other when prescribing.

The current research found 4 advantages of DJD in treating RA compared to rDMARDs. First, DJD treatment could better improve the laboratory indicators, including the level of RF, T lymphocyte subpopulation (including CD4^+^, CD8^+^, and CD4^+^/CD8^+^), and inflammatory biomarkers (including ESR, CRP, TNF-α, IL-6, IL-1β, and IL-1) compared to rDMARDs.

Pre-clinical RA is typically characterized by subclinical inflammation, manifested by elevated levels of serum CRP and other pro-inflammatory cytokines and chemokines.^[73–75]^ Many scholars hold the view that anti-citrullinated protein antibodies (ACPAs) are highly specific for RA and closely related to the pathogenesis of the disease.^[76,77]^ The amplification of ACPAs could promote the subsequent increase of the concentration of inflammatory cytokines in serum, including TNF, IL-6, etc.^[78,79]^ In addition, according to the magnetic resonance imaging results of RA, there is a stable correlation between joint inflammation and systemic inflammatory indicators, such as CRP, ESR, and IL-6.^[73,80,81]^ Furthermore, the pathological process of RA can be driven by inflammatory cascade reactions, including excessive production and expression of IL-1, IL-17, and TNF-α.^[82–85]^ In the same vein, it was found that IL-1β and TNF-α can induce RA progression through mediators such as cyclooxygenase-2 and matrix metalloproteinases, and cyclooxygenase-2 can increase the production of prostaglandin E2, ultimately leading to synovial inflammation.^[86,87]^ There is a large volume of published studies describing that many active ingredients in DJD could decrease the levels of inflammatory factors mentioned above. One study by Leng et al found that naringenin, one of active ingredient in DJD, can reduce the levels of RF, CRP, and IL-17 in serum as well as the infiltration of inflammatory cells in RA rats.^[88]^ Shi et al held the view that formononetin, an active component in DJD, can decrease the levels of TNF-α and IL-6 in serum and the expression of NF-κB, p65, p-p65, and PCNP proteins in spleen tissue.^[89]^ Recently, in vitro and in vivo studies have shown that columbianadin, one of the main bioactive compounds of *Radix Angelicae Pubescentis* (Du Huo), could inhibit inflammatory response, regulate oxidative stress, and improve changes in gut microbiota and metabolites by regulating JAK1/STAT3, NF-κB, and Keap1/Nrf2 signaling pathways in CIA mice.^[90]^ Similarly, it was found that columbianadin can inhibit the secretion of the inflammatory cytokine IL-17A, promote its differentiation towards treg cells, and promote the secretion of anti-inflammatory cytokines.^[91]^ Several studies have revealed that osthole, an active ingredients of *Radix Angelicae Pubescentis* (Du Huo), can inhibit the proliferation, migration, and invasion of RA fibroblast like synovial cells by weakening NF-κB signaling,^[92]^ and activate AMPK to inhibit NLRP3 inflammasome activation.^[93]^ Additionally, many inflammatory factors, including IL-1β, IL-6, TNF-α, etc., were found to be decreased by active ingredients or extracts in DJD in both in vivo and in vitro experiments of RA, such as senkyunolide I,^[94]^ 18β-Glycyrrhetinic acid,^[95]^ liquiritin,^[96]^ polysaccharide from *Radix Angelicae sinensis* (Dang Gui),^[97]^ paeoniflorin,^[98–102]^ paeoniflorin-6’-O-benzene sulfonate,^[103]^ curcumin,^[104]^ the total saponins of *Radix Achyranthis bidentatae* (Niu Xi),^[105]^ supercritical carbon dioxide extraction of *Radix Achyranthis bidentatae* (Niu Xi),^[106]^ polysaccharide from *Radix Saposhnikoviae divaricatae* (Fang Feng),^[107,108]^ and *Radix Saposhnikoviae divaricatae* (Fang Feng).^[109]^ The current research found that the level of inflammatory biomarkers, including ESR, CRP, TNF-α, IL-6, IL-1β, and IL-1, could be better lowered by DJD treatment than rDMARDs. It is worth noting that, according to a few subgroup analysis results, there were no significant differences in the reduction of some inflammatory biomarker levels between DJD and rDMARDs. For example, when the experimental group only used DJD instead of DJD combined with rDMARDs, there was no significant difference in IL-6 between the experimental group and the control group. Nonetheless, the reliability of our conclusions may not be affected as only 2 studies reported results in this regard.

RF is anti-IgG autoantibodies strongly associated with RA.^[110]^ And it could activate complement through classical pathways, leading to pathogenic outcomes involving inflammatory events and tissue damage.^[110,111]^ One study by Ganova, Gyurkovska, Belenska-Todorova and Ivanovska suggested that there was local complement synthesis and consumption in the joints of patients with RA.^[112]^ According to Scott, Wolfe and Huizinga,^[113]^ RF levels in patients who respond to treatment often decrease and rise again in clinical recurrence. A previous study found that naringenin could reduce RF levels in vivo by regulating the Treg/Th17 balance.^[88]^ Furthermore, in vivo studies have shown that the level of RF can be significantly decreased by DJD and aconitum extract hydrogel.^[114,115]^ Therefore, according to the results of this aspect, we hold the view that the level of RF could be better reduced by DJD than rDMARDs.

The main inflammatory site of RA is the synovial membrane of the joint.^[116]^ There is a group of T lymphocytes in RA synovial tissue that can form lymph node-like tissue structures, and these T cell clusters are composed of small and oligocytoplasmic CD4^+^memory T (CD45RO^+^) cells.^[117,118]^ Studies have shown that the infiltration of T cells in synovial tissue is one of the important features of this disease, and multiple T lymphocytes are involved in the pathogenesis of RA.^[116,119,120]^ It was found that columbianadin can inhibit the differentiation of CD4 T lymphocytes towards th17 cells and promote their differentiation towards treg cells in CIA mice and the spleen lymphocytes of balb/c mice.^[91]^ Our meta-analysis indicated that DJD treatment could better improve the level of CD4^+^, CD8^+^, and CD4^+^/CD8^+^ than rDMARDs. However, further research is necessary to confirm the efficacy of these aspects, as limited data (including 140 patients in the treatment and control groups) may lead to false positive results.

Second, compared to rDMARDs, DJD could better improve the main symptoms and signs of RA, including the duration of morning stiffness, the number of joint tenderness, the number of swollen joints, and the grip strength of both hands. The symptoms and signs of RA are mainly swelling, stiffness, and pain in joints caused by active synovitis.^[121]^ Morning stiffness, joint tenderness, and joint swelling are important diagnostic criteria and treatment targets for RA.^[113,121]^ Additionally, RA is one of the recognized causes of systemic bone loss, which means that patients have a significantly increased risk of osteoporosis and fractures.^[122–124]^ Previous evidence suggests that one of the risk factors for bone loss and fractures in RA includes decreased grip strength.^[125–127]^ This is because the systemic inflammation of RA leads to an increase in the expression of growth factors, thus activating receptors, and promoting osteoclast differentiation and bone resorption.^[128–130]^ A recent study found that the degree of arthritis swelling in CIA model rats could be ameliorated by ingredients in DJD, including isoimperatorin, zosimin, and isopsoralen.^[131]^ It was found that osthole can significantly improve joint deformities and toe erythema in CIA rats, possibly by regulating mitochondrial homeostasis and function.^[93]^ Shi et al held the view that formaronetin can reduce the degree of paw swelling in RA mice, which may be achieved by regulating the NF-κB p65 signaling pathway and inhibiting the expression of inflammatory factors.^[89]^ Similarly, previous studies found that the degree of paw swelling could be relieved by active ingredients in DJD, such as columbianadin,^[90]^ the total saponins of *Radix Achyranthis bidentatae* (Niu Xi),^[105]^ polysaccharide from *Radix Angelicae sinensis* (Dang Gui),^[97]^ and paeoniflorin.^[100,102]^ Our meta-analysis showed that the main symptoms and signs of RA could be better improved by DJD treatment than rDMARDs. However, the results of some subgroup analyses are worth noting. For instance, when the experimental group only used DJD instead of DJD combined with rDMARDs, there was no significant difference between the experimental group and the control group in terms of the duration of morning stiffness, the number of joint tenderness, and the number of swollen joints. Furthermore, in the aspect of the grip strength of both hands, there was no significant difference when the age was less than 47 years old, the course of disease was greater than or equal to 4 years, the course of treatment was less than 12 weeks, etc. Therefore, although the results of most subgroup analysis were consistent with the overall research results, these inconsistent subgroup analysis results should be treated with caution and further verified.

Third, compared to rDMARDs, DJD treatment could better improve questionnaire scores in RA patients, including VAS, HAQ, and DAS-28. In clinical trials of RA, these questionnaires are often used to evaluate treatment effectiveness.^[132–134]^ One study by Guo et al revealed that naringenin can reduce the joint lesion score of RA rats, which may be achieved by regulating Treg/Th17 balance.^[88]^ In the same vein, several studies have revealed that the joint lesion score could be ameliorated by columbianadin,^[90]^ osthole,^[93]^ and liquiritin.^[96]^ According to our meta-analysis results, we concluded that the score of VAS, HAQ, and DAS-28 could be better improved by DJD treatment than rDMARDs. However, part of the subgroup analysis results differed from the overall results. For example, when the age was greater than or equal to 47 years old, there was no significant difference between the experimental group and the control group in terms of VAS, HAQ, and DAS-28; when the course of disease was less than 4 years, there was no significant difference between the experimental group and the control group in terms of HAQ and DAS-28. Therefore, when using these questionnaires, attention should be paid to conducting subgroup analysis to avoid false positive results affecting the evaluation of efficacy. Overall, in most cases, VAS, HAQ, and DAS-28 scores could be better improved by DJD treatment than rDMARDs.

Fourth, DJD treatment could be safer than rDMARDs. Adverse events were reported in 22 studies, which include abnormal liver function, decreased white blood cells, rash, hair loss, dizziness, nausea, vomiting, bloating, diarrhea, etc. Most symptoms were mild, tolerable, and could be relieved automatically. One study by Feng et al found that 18β-Glycyrrhetinic acid could mitigate liver damage caused by collagen or MTX.^[95]^ Similarly, Aggarwal et al held the view that curcumin is highly safe for humans.^[104]^ The present study suggests that patients treated with DJD had fewer adverse events than rDMARDs. Interestingly, according to the subgroup analysis results, there was no significant difference in AEs between the experimental group and the control group when the experimental group only used DJD instead of DJD combined with rDMARDs. Nevertheless, this result should be treated with caution as only 2 studies reported this result. In conclusion, DJD treatment, especially DJD combined with rDMARDs, could be safer than rDMARDs.

### 4.2. Source of heterogeneity

Due to the high heterogeneity of some results, we searched for the source of heterogeneity through subgroup analysis, meta-regression, and sensitivity analysis. Subgroup analysis and meta-regression were conducted based on age, course of disease, course of treatment, interventions used in the experimental or control group, and random sequence generation. For some indicators, heterogeneity was decreased after subgroup analysis. We concluded that, according to the results of meta-regression and subgroup analysis, 4 aspects may be the reasons for the high heterogeneity, including age, interventions used in the experimental or control group, and random sequence generation. Especially interventions used in the experimental or control group were the main reason for the high heterogeneity. Furthermore, based on the sensitivity analysis results, some included studies may also be the cause of high heterogeneity. We found that heterogeneity was significantly decreased when these studies were excluded.

Another reason for the heterogeneity may be the unclear risk of bias. In 42 included studies, only 26 described detailed randomization methods. And all allocation concealment and blinding of outcome assessment of included studies were evaluated as unclear risk. In terms of blinding of participants and personnel, only one study was evaluated as low risk. These could also bring heterogeneity. Lastly, the complexity of RA, etiology, disease history, nursing treatment, rDMARDs strategies, and herbs origin may all contribute to heterogeneity. Taken together, the high heterogeneity may be mainly caused by interventions used in the experimental or control group, as well as some included studies.

### 4.3. Agreement and disagreement with previous studies

One study by Zhang, Wang, Zhu, Tian and Xiang conducted a meta-analysis on the effectiveness of DJD in treating RA, including 7 outcome indicators from 22 studies.^[135]^ Compared with them, we included 19 outcome indicators from 42 studies. Their 7 outcome indicators were included in our meta-analysis, including effective rate, ESR, CRP, RF, duration of morning stiffness, number of joint tenderness, and number of swollen joints. Our meta-analysis was inconsistent with them only in terms of effective rate, that is, we found that there was no significant difference between DJD treatment and rDMARDs. Although we speculated that this finding may be due to different efficacy criteria and conducted corresponding subgroup analysis, the results of the subgroup analysis were consistent with our overall research results. For their other 6 outcomes, we agree with their viewpoint that DJD treatment was better than rDMARDs. In their study, no meta-analysis was conducted on the AEs, thus they fail to draw any conclusion about the safety of DJD for RA. In our study, we have included AEs and conducted a series of related analyses. The results showed that DJD, especially DJD combined with rDMARDs, could be safer than rDMARDs. In addition, we conducted subgroup analysis, meta-regression, and sensitivity analysis to identify possible sources of heterogeneity, which were not conducted in their study. In terms of publication bias assessment, they only analyzed the effective rate and believed that there was no publication bias through the funnel plot. However, we believed that there was a publication bias in the effective rate according to Egger’s test and funnel plot. In addition, we also tested for publication bias on the other 10 outcome indicators. In summary, compared to the previous study, we had a larger sample size, more comprehensive outcome indicators, and more detailed analysis to explore both the efficacy and safety of DJD for RA.

### 4.4. Strengths and limitations of this study

Our study included the following strengths. First, we included a large number of RCTs and outcome indicators related to RA, including 19 outcome indicators of 3635 patients in 42 articles. Due to the relatively sufficient number of original studies, we also conducted subgroup analysis on 13 outcome indicators to observe whether the data after subgroup analysis was consistent with the overall results. Second, we conducted subgroup analysis, meta-regression, and sensitivity analysis to explore possible sources of heterogeneity, such as age, course of disease, course of treatment, interventions used in the experimental or control group, random sequence generation, or some original research. These results provided a comprehensive summary of relevant information to support the efficacy and safety of DJD in RA. Therefore, these could provide a new idea and direction for clinical treatment.

However, several limitations of this study need to be considered. First, there is a risk of bias due to the lack of blinding and unclear randomization methods. The low quality of the included research may weaken the credibility of the results. Second, some results exhibit high heterogeneity. Although most significant heterogeneity was decreased after subgroup analysis, meta-regression, or sensitivity analysis, there were still some sources of heterogeneity that cannot be fully determined. The heterogeneity may be caused by various factors, such as the severity of diseases, the composition and dosage of TCM prescriptions, or the included study itself. Third, due to the characteristics of syndrome differentiation and treatment in TCM, the DJD used in some original studies was slightly modified, which may limit the widespread application of the results. In conclusion, although our study indicated that DJD exhibits good potential in treating RA, further research is needed to address these limitations. Therefore, more high-quality RCTs are needed to provide sufficient information for long-term research on the efficacy and safety of DJD in the treatment of RA.

### 4.5. Implications for future research

Based on the above findings and limitations, the following suggestions are provided for future practice. First, the rigor of research plans should be improved, and protocols should be registered in advance to ensure information transparency. Although the blind methods in TCM are not easy to implement, the generation of random sequences should be taken seriously. Second, research information should be recorded in detail for subgroup analysis, such as data related to gender, age, course of disease, disease severity, treatment duration, and dosage. Third, long-term follow-up studies should be encouraged to elucidate the efficacy of DJD in laboratory indicators, symptoms, and signs of RA patients. Finally, the material basis and mechanism of DJD treatment for RA patients should be revealed at the cellular, molecular, and genetic levels. This may help identify high-risk patients with these situations and allow for earlier and more effective interventions.

## 5. Conclusion

The evidence from this study suggests that DJD could be a satisfactory complementary and alternative therapy for the treatment of RA. Compared with rDMARDs, DJD treatment could better improve the level of laboratory indicators, main symptoms and signs, and some questionnaire scores of RA patients with fewer adverse events. The laboratory indicators included RF, CD4^+^, CD8^+^, CD4^+^/CD8^+^, ESR, CRP, TNF-α, IL-6, IL-1β, and IL-1; The main symptoms and signs included the duration of morning stiffness, the number of joint tenderness, the number of swollen joints, and the grip strength of both hands; and the questionnaires included VAS, HAQ, and DAS-28. However, given the low quality of the included studies and the small number of subgroup analysis results being different from the overall results, further research is needed to improve the scientific credibility of the evidence.

## Acknowledgments

We would like to thank the project of first-class discipline construction in Yunnan Province for its support in this study, and acknowledge Junjie Zhou, Jiana Hui, Rongfang Zhang, Lingru Yang, and Mengfei Wang of the Yunnan University of Chinese Medicine for assistance in data entry.

## Author contributions

**Conceptualization:** Pengda Qu, Xiaohu Tang.

**Data curation:** Pengda Qu, Wei Wang, Shiyu Du.

**Formal analysis:** Pengda Qu.

**Funding acquisition:** Pengda Qu, Xiaohu Tang.

**Investigation:** Haiyang Wang, Wei Wang, Shiyu Du, Qian Hu.

**Methodology:** Haiyang Wang, Wei Wang, Zhaorong Peng, Qian Hu, Xiaohu Tang.

**Project administration:** Xiaohu Tang.

**Resources:** Zhaorong Peng, Xiaohu Tang.

**Software:** Pengda Qu, Haiyang Wang, Zhaorong Peng, Qian Hu.

**Supervision:** Zhaorong Peng, Xiaohu Tang.

**Validation:** Shiyu Du.

**Writing – original draft:** Pengda Qu, Wei Wang, Qian Hu.

**Writing – review & editing:** Pengda Qu.

## Supplementary Material
