# Supplementary material for: Efficacy and safety of Duhuo-Jisheng decoction in rheumatoid arthritis: A systematic review and meta-analysis of 42 randomized controlled trials
Source: Medicine (Baltimore). 2023 Nov 3;102(44):e35513. doi: 10.1097/MD.0000000000035513 (PMC10627613; doi:10.1097/MD.0000000000035513)
Supplement: Supplementary file 1 [file medi-102-e35513-s001.docx]

Supplemental Table S1. The specific search strategy of PubMed.

| **Number** | **Search terms** |
| --- | --- |
| #1 | "Arthritis, Rheumatoid"[Mesh] OR Arthritis, Rheumatoid [All Fields] OR Rheumatoid Arthritis [All Fields] OR Rheumatic Arthritis [All Fields] OR Arthritis, Rheumatic [All Fields] OR Rheumatism, Articular [All Fields] OR Polyarthritis Rheumatica [All Fields] OR Polyarthritis Rheumaticas [All Fields] OR Rheumatic Arthritides [All Fields] OR Articular Rheumatism [All Fields] OR Articular Rheumatisms [All Fields] |
| #2 | (Duhuojishengtang [All Fields] OR Duhuo-Jisheng-Tang [All Fields] OR Duhuojisheng Decoction [All Fields] OR Duhuo-Jisheng Decoction [All Fields] OR Duhuo-Jisheng [All Fields]) OR |
| #3 | (Duhuo [All Fields] OR Du Huo [All Fields] OR Radix Angelicae Pubescentis [All Fields] OR Doubleteeth Pubescent Angelica Root [All Fields]) AND (Sangjisheng [All Fields] OR Shang Ji Sheng [All Fields] OR Herba Taxilli [All Fields] OR Chinese Taxillus Herb) AND (Duzhong [All Fields] OR Du Zhong [All Fields] OR Eucommia bark [All Fields] OR Cortex Eucommiae [All Fields]) AND (Niuxi [All Fields] OR Niu Xi [All Fields] OR root of Twotooth Achyranthes [All Fields] OR Radix Achyranthis bidentatae [All Fields]) AND (Xixin [All Fields] OR Xi Xin [All Fields] OR all-grass Manchurian wildginger [All Fields] OR Herba Asari [All Fields]) AND (Qinjiao [All Fields] OR Qin Jiao [All Fields] OR root of Largeleaf Gentian [All Fields] OR Radix Gentiae macrophyllae [All Fields]) AND (Fuling [All Fields] OR Fu Ling [All Fields] OR Indian Bread [All Fields] OR Poria [All Fields]) AND (Rougui [All Fields] OR Rou Gui [All Fields] OR Cassia Bark [All Fields] OR Cortex Cinmomi [All Fields]) AND (Fangfeng [All Fields] OR Fang Feng [All Fields] OR root of Divaricate Saposhnikovia [All Fields] OR Radix Saposhnikoviae divaricatae [All Fields]) AND (Chuanxiong [All Fields] OR Chuan Xiong [All Fields] OR Chuanxiong rhizome, Szechuan lovage root [All Fields] OR Radix chuanxiong [All Fields] OR Rhizoma Chuanxiong [All Fields]) AND (Renshen [All Fields] OR Ren Shen [All Fields] OR Ginseng [All Fields] OR Radix Ginseng [All Fields]) AND (Gancao [All Fields] OR Gan Cao [All Fields] OR Root of Ural Licorice [All Fields] OR Radix Glycyrrhizae [All Fields]) AND (Danggui[All Fields] OR Dang Gui [All Fields] OR root of Chinese Angelica [All Fields] OR Radix Angelicae sinensis [All Fields]) AND (Baishao [All Fields] OR Bai Shao[All Fields] OR Shaoyao [All Fields] OR Shao Yao [All Fields] OR White peony root [All Fields] OR Radix Paeoniae Alba [All Fields]) AND (Gandihuang [All Fields] OR Gan Di Huang [All Fields] OR Adhesive Rehmannia Dried Root [All Fields] OR Rehmannia glutinosa [All Fields]) |
| #4 | #1 AND (#2 OR #3) |

**Article title:** Efficacy and safety of Duhuo-Jisheng decoction in rheumatoid arthritis: A systematic review and meta-analysis of 42 randomized controlled trials. **First author**: Pengda Qu
