# Supplementary material for: Efficacy and safety of Duhuo-Jisheng decoction in rheumatoid arthritis: A systematic review and meta-analysis of 42 randomized controlled trials
Source: Medicine (Baltimore). 2023 Nov 3;102(44):e35513. doi: 10.1097/MD.0000000000035513 (PMC10627613; doi:10.1097/MD.0000000000035513)
Supplement: Supplementary file 2 [file medi-102-e35513-s002.docx]

Supplemental Table S2. The results of subgroup analysis.

| Outcomes or subgroup | Number of studies | Results WMD/RR/RD (95% CI) | Q | *P*−value for overall effect | I^2^(%) | *P*−value for heterogeneity between groups | *P*−value for meta-regression |
| --- | --- | --- | --- | --- | --- | --- | --- |
| 1. Effective rate | 38 | 0.18 [0.15, 0.21] | 45.53 | 0.16 | 18.74 | / | / |
| Age  Greater than or equal to 47 years old  Less than 47 years old | 38  20  18 | 0.18 [0.15, 0.21]  0.20 [0.16, 0.24]  0.16 [0.12, 0.20] | 45.53  17.37  27.03 | 0.16  0.56  0.06 | 18.74  0.00  37.11 | 0.21 | / |
| Course of disease  Greater than or equal to four years  Less than four years | 35  18  17 | 0.18 [0.15, 0.21]  0.18 [0.13, 0.22]  0.19 [0.14, 0.23] | 44.55  24.61  20.34 | 0.11  0.10  0.21 | 23.68  30.93  21.32 | 0.72 | / |
| Course of treatment  Greater than or equal to 12 weeks  Less than 12 weeks | 38  15  23 | 0.18 [0.15, 0.21]  0.20 [0.15, 0.24]  0.18 [0.14, 0.21] | 45.53  25.42  19.68 | 0.16  0.03  0.60 | 18.74  44.93  0.00 | 0.54 | / |
| Experimental group  DJD  DJD + C | 38  6  32 | 0.18 [0.15, 0.21]  0.49 [0.10, 0.27]  0.18 [0.15, 0.21] | 45.53  3.78  41.64 | 0.16  0.58  0.10 | 18.74  0.00  25.55 | 0.93 | / |
| Control group  Multiple drugs  Single drug | 38  20  18 | 0.18 [0.15, 0.21]  0.19 [0.14, 0.23]  0.18 [0.14, 0.22] | 45.53  26.30  18.89 | 0.16  0.09  0.40 | 18.74  31.55  4.72 | 0.77 | / |
| Random sequence generation  Low risk  Unclear risk | 38  23  15 | 0.18 [0.15, 0.21]  0.19 [0.15, 0.23]  0.18 [0.13, 0.22] | 45.53  26.12  18.50 | 0.16  0.25  0.19 | 18.74  15.77  24.31 | 0.64 | / |
| 2. Erythrocyte sedimentation rate (ESR) | 29 | -11.96 [-14.28, -9.64] | 119.62 | 0.00 | 76.59 | / | / |
| Age  Greater than or equal to 47 years old  Less than 47 years old | 29  14  15 | -11.96 [-14.28, -9.64]  -13.28 [-16.99, -9.56]  -10.85 [-13.68, -8.01] | 119.62  42.26  61.25 | 0.00  0.00  0.00 | 76.59  69.24  77.14 | 0.31 | 0.79 |
| Course of disease  Greater than or equal to four years  Less than four years | 26  12  14 | -11.77 [-14.28, -9.25]  -11.71 [-15.08, -8.34]  -11.75 [-15.91, -7.59] | 103.46  53.83  49.56 | 0.00  0.00  0.00 | 75.84  79.57  73.77 | 0.99 | 0.49 |
| Course of treatment  Greater than or equal to 12 weeks  Less than 12 weeks | 29  13  16 | -11.96 [-14.28, -9.64]  -12.37 [-16.68, -8.07]  -11.79 [-14.54, -9.04] | 119.62  68.09  51.48 | 0.00  0.00  0.00 | 76.59  82.38  70.86 | 0.82 | 0.80 |
| Experimental group  DJD  DJD + C | 29  3  26 | -11.96 [-14.28, -9.64]  -10.01 [-20.09, 0.08]  -11.96 [-14.28, -9.64] | 119.62  11.96  107.59 | 0.00  0.00  0.00 | 76.59  83.28  76.76 | 0.69 | 0.49 |
| Control group  Multiple drugs  Single drug | 29  12  17 | -11.96 [-14.28, -9.64]  -8.93 [-11.66, -6.19]  -13.49 [-16.74, -10.24] | 119.62  24.75  76.86 | 0.00  0.01  0.00 | 76.59  55.56  79.18 | 0.04 | 0.02 |
| Random sequence generation  Low risk  Unclear risk | 29  16  13 | -11.96 [-14.28, -9.64]  -12.87 [-15.76, -9.98]  -10.61 [-14.77, -6.46] | 119.62  61.30  57.02 | 0.00  0.00  0.00 | 76.59  75.53  78.95 | 0.38 | 0.07 |
| 3. C-reactive protein (CRP) | 28 | -6.43 [-7.75, -5.11] | 135.41 | 0.00 | 80.06 | / | / |
| Age  Greater than or equal to 47 years old  Less than 47 years old | 28  13  15 | -6.43 [-7.75, -5.11]  -5.63 [-7.15, -4.10]  -7.14 [-9.29, -4.98] | 135.41  31.33  103.32 | 0.00  0.00  0.00 | 80.06  61.69  86.45 | 0.26 | 0.21 |
| Course of disease  Greater than or equal to four years  Less than four years | 25  12  13 | -5.73 [-7.04, -4.43]  -6.61 [-8.92, -4.30]  -5.18 [-6.77, -3.58] | 110.03  69.16  40.13 | 0.00  0.00  0.00 | 78.19  84.10  70.10 | 0.32 | 0.24 |
| Course of treatment  Greater than or equal to 12 weeks  Less than 12 weeks | 28  12  16 | -6.43 [-7.75, -5.11]  -7.03 [-9.68, -4.38]  -6.42 [-8.06, -4.78] | 135.41  44.47  90.89 | 0.00  0.00  0.00 | 80.06  75.27  83.50 | 0.70 | 0.35 |
| Experimental group  DJD  DJD + C | 28  3  25 | -6.43 [-7.75, -5.11]  -2.85 [-4.68, -1.03]  -7.19 [-8.65, -5.74] | 135.41  8.52  90.11 | 0.00  0.01  0.00 | 80.06  76.53  73.37 | 0.00 | 0.00 |
| Control group  Multiple drugs  Single drug | 28  12  16 | -6.43 [-7.75, -5.11]  -4.46 [-5.19, -3.74]  -8.07 [-10.19, -5.95] | 135.41  8.93  126.27 | 0.00  0.63  0.00 | 80.06  0.00  88.12 | 0.00 | 0.04 |
| Random sequence generation  Low risk  Unclear risk | 28  17  11 | -6.43 [-7.75, -5.11]  -5.86 [-7.05, -4.66]  -9.11 [-13.15, -5.08] | 135.41  54.12  59.95 | 0.00  0.00  0.00 | 80.06  70.44  83.32 | 0.13 | 0.51 |
| 4. Rheumatoid factor (RF) | 25 | -24.58 [-30.13, -19.03] | 211.01 | 0.00 | 88.63 | / | / |
| Age  Greater than or equal to 47 years old  Less than 47 years old | 25  13  12 | -24.58 [-30.13, -19.03]  -21.90 [-28.46, -15.34]  -29.86 [-41.05, -18.67] | 211.01  105.18  93.42 | 0.00  0.00  0.00 | 88.63  88.59  88.23 | 0.23 | 0.22 |
| Course of disease  Greater than or equal to four years  Less than four years | 22  11  11 | -22.27 [-27.85, -16.69]  -35.71 [-49.42, -22.01]  -10.80 [-14.39, -7.20] | 190.30  131.63  26.69 | 0.00  0.00  0.00 | 88.96  92.40  62.53 | 0.00 | 0.07 |
| Course of treatment  Greater than or equal to 12 weeks  Less than 12 weeks | 25  12  13 | -24.58 [-30.13, -19.03]  -32.37 [-42.79, -21.94]  -16.07 [-21.99, -10.15] | 211.01  165.01  42.33 | 0.00  0.00  0.00 | 88.63  93.33  71.65 | 0.01 | 0.72 |
| Experimental group  DJD  DJD + C | 25  1  24 | -24.58 [-30.13, -19.03]  -5.80 [-14.91, 3.31]  -25.93 [-31.72, -20.14] | 211.01  -0.00  209.00 | 0.00  /  0.00 | 88.63  /  89.00 | 0.00 | 0.97 |
| Control group  Multiple drugs  Single drug | 25  10  15 | -24.58 [-30.13, -19.03]  -17.15 [-25.45, -8.84]  -32.29 [-41.48, -23.09] | 211.01  91.86  110.62 | 0.00  0.00  0.00 | 88.63  90.20  87.34 | 0.00 | 0.27 |
| Random sequence generation  Low risk  Unclear risk | 25  15  10 | -24.58 [-30.13, -19.03]  -17.65 [-23.09, -12.22]  -34.84 [-44.96, -24.72] | 211.01  108.88  22.24 | 0.00  0.00  0.01 | 88.63  87.14  59.53 | 0.00 | 0.08 |
| 5. Tumor necrosis factor-α (TNF-α) | 13 | -26.35 [-36.54, -16.16] | 825.58 | 0.00 | 98.55 | / | / |
| Age  Greater than or equal to 47 years old  Less than 47 years old | 13  8  5 | -26.35 [-36.54, -16.16]  -19.58 [-31.17, -7.99]  -37.10 [-48.16, -26.05] | 825.58  319.71  24.29 | 0.00  0.00  0.00 | 98.55  97.81  83.53 | 0.03 | 0.44 |
| Course of disease  Greater than or equal to four years  Less than four years | 11  7  4 | -23.50 [-34.31, -12.70]  -21.29 [-34.45, -8.14]  -27.40 [-47.18, -7.62] | 769.27  580.82  84.17 | 0.00  0.00  0.00 | 98.70  98.97  96.44 | 0.61 | 0.37 |
| Course of treatment  Greater than or equal to 12 weeks  Less than 12 weeks | 13  8  5 | -26.35 [-36.54, -16.16]  -21.40 [-33.66, -9.14]  -34.76 [-54.52, -14.99] | 825.58  623.41  85.35 | 0.00  0.00  0.00 | 98.55  98.88  95.31 | 0.26 | 0.95 |
| Experimental group  DJD  DJD + C | 13  1  12 | -26.35 [-36.54, -16.16]  -2.62 [-9.96, 4.72]  -28.41 [-39.24, -17.58] | 825.58  0.00  822.63 | 0.00  /  0.00 | 98.55  /  98.66 | 0.00 | 0.60 |
| Control group  Multiple drugs  Single drug | 13  5  8 | -26.35 [-36.54, -16.16]  -20.22 [-36.91, -3.54]  -30.36 [-44.10, -16.62] | 825.58  563.86  161.99 | 0.00  0.00  0.00 | 98.55  99.29  95.68 | 0.36 | 0.46 |
| Random sequence generation  Low risk  Unclear risk | 13  10  3 | -26.35 [-36.54, -16.16]  -25.05 [-36.36, -13.74]  -31.88 [-40.85, -22.91] | 825.58  697.64  4.92 | 0.00  0.00  0.09 | 98.55  98.71  59.33 | 0.35 | 0.62 |
| 6. Interleukin 6 (IL-6) | 12 | -8.40 [-11.00, -5.81] | 205.27 | 0.00 | 94.64 | / | / |
| Age  Greater than or equal to 47 years old  Less than 47 years old | 12  9  3 | -8.40 [-11.00, -5.81]  -6.66 [-8.89, -4.43]  -15.35 [-24.84, -5.86] | 205.27  86.74  64.99 | 0.00  0.00  0.00 | 94.64  90.78  96.92 | 0.08 | 0.00 |
| Course of disease  Greater than or equal to four years  Less than four years | 11  6  5 | -8.84 [-11.66, -6.02]  -10.90 [-16.60, -5.20]  -7.07 [-8.80, -5.34] | 203.91  176.84  12.32 | 0.00  0.00  0.02 | 95.10  97.17  67.54 | 0.21 | 0.05 |
| Course of treatment  Greater than or equal to 12 weeks  Less than 12 weeks | 12  7  5 | -8.40 [-11.00, -5.81]  -7.36 [-10.68, -4.03]  -10.51 [-15.57, -5.44] | 205.27  143.89  44.75 | 0.00  0.00  0.00 | 94.64  95.83  91.06 | 0.31 | 0.09 |
| Experimental group  DJD  DJD + C | 12  2  10 | -8.40 [-11.00, -5.81]  -7.52 [-14.27, -0.77]  -8.55 [-11.35, -5.76] | 205.27  2.17  201.15 | 0.00  0.14  0.00 | 94.64  53.90  95.53 | 0.78 | 0.05 |
| Control group  Multiple drugs  Single drug | 12  5  7 | -8.40 [-11.00, -5.81]  -6.78 [-10.91, -2.65]  -9.67 [-12.88, -6.45] | 205.27  128.86  45.43 | 0.00  0.00  0.00 | 94.64  96.90  86.79 | 0.28 | 0.02 |
| Random sequence generation  Low risk  Unclear risk | 12  11  1 | -8.40 [-11.00, -5.81]  -8.48 [-11.39, -5.57]  -8.27 [-9.67, -6.87] | 205.27  190.68  0.00 | 0.00  0.00  / | 94.64  94.76  / | 0.90 | 0.03 |
| 7. Duration of morning stiffness | 25 | -17.46 [-21.06, -13.86] | 149.38 | 0.00 | 83.93 | / | / |
| Age  Greater than or equal to 47 years old  Less than 47 years old | 25  11  14 | -17.46 [-21.06, -13.86]  -17.38 [-24.13, -10.64]  -17.79 [-22.10, -13.48] | 149.38  71.22  66.94 | 0.00  0.00  0.00 | 83.93  85.96  80.58 | 0.92 | 0.73 |
| Course of disease  Greater than or equal to four years  Less than four years | 22  11  11 | -17.14 [-21.18, -13.10]  -18.11 [-24.37, -11.84]  -16.25 [-22.29, -10.21] | 147.32  56.67  90.05 | 0.00  0.00  0.00 | 85.74  82.35  88.90 | 0.68 | 0.78 |
| Course of treatment  Greater than or equal to 12 weeks  Less than 12 weeks | 25  10  15 | -17.46 [-21.06, -13.86]  -16.93 [-24.62, -9.24]  -17.58 [-21.79, -13.37] | 149.38  67.92  79.47 | 0.00  0.00  0.00 | 83.93  86.75  82.38 | 0.88 | 0.96 |
| Experimental group  DJD  DJD + C | 25  3  22 | -17.46 [-21.06, -13.86]  -19.16 [-29.74, -8.58]  -17.25 [-21.13, -13.38] | 149.38  5.44  143.94 | 0.00  0.07  0.00 | 83.93  86.75  85.41 | 0.74 | 0.74 |
| Control group  Multiple drugs  Single drug | 25  11  14 | -17.46 [-21.06, -13.86]  -21.26 [-25.40, -17.13]  -14.64 [-20.16, -9.12] | 149.38  40.87  73.20 | 0.00  0.00  0.00 | 83.93  75.53  82.24 | 0.06 | 0.16 |
| Random sequence generation  Low risk  Unclear risk | 25  15  10 | -17.46 [-21.06, -13.86]  -19.31 [-23.34, -15.28]  -14.07 [-21.87, -6.27] | 149.38  62.59  86.77 | 0.00  0.00  0.00 | 83.93  77.63  89.63 | 0.24 | 0.33 |
| 8. Number of joint tenderness | 18 | -2.42 [-2.99, -1.84] | 50.39 | 0.00 | 66.26 | / | / |
| Age  Greater than or equal to 47 years old  Less than 47 years old | 18  9  9 | -2.42 [-2.99, -1.84]  -2.21 [-3.06, -1.35]  -2.67 [-3.53, -1.82] | 50.39  26.82  23.26 | 0.00  0.00  0.00 | 66.26  70.18  65.61 | 0.45 | 0.56 |
| Course of disease  Greater than or equal to four years  Less than four years | 16  9  7 | -2.27 [-2.86, -1.69]  -2.14 [-2.81, -1.48]  -2.42 [-3.56, -1.27] | 44.52  17.93  24.52 | 0.00  0.02  0.00 | 66.31  55.39  75.53 | 0.68 | 0.75 |
| Course of treatment  Greater than or equal to 12 weeks  Less than 12 weeks | 18  9  9 | -2.42 [-2.99, -1.84]  -2.20 [-3.12, -1.28]  -2.54 [-3.29, -1.78] | 50.39  11.94  37.96 | 0.00  0.06  0.00 | 66.26  49.74  73.66 | 0.58 | 0.88 |
| Experimental group  DJD  DJD + C | 18  2  16 | -2.42 [-2.99, -1.84]  -2.29 [-4.16, -0.43]  -2.42 [-2.99, -1.84] | 50.39  2.00  48.16 | 0.00  0.16  0.00 | 66.26  49.92  68.85 | 0.89 | 0.95 |
| Control group  Multiple drugs  Single drug | 18  8  10 | -2.42 [-2.99, -1.84]  -2.53 [-3.43, -1.63]  -2.33 [-3.15, -1.52] | 50.39  27.30  22.43 | 0.00  0.00  0.01 | 66.26  74.36  59.88 | 0.75 | 0.51 |
| Random sequence generation  Low risk  Unclear risk | 18  11  7 | -2.42 [-2.99, -1.84]  -2.47 [-3.11, -1.84]  -2.27 [-3.55, -0.99] | 50.39  25.73  24.14 | 0.00  0.00  0.00 | 66.26  61.14  75.14 | 0.78 | 0.96 |
| 9. Number of swollen joints | 18 | -1.87 [-2.53, -1.21] | 113.60 | 0.00 | 87.28 | / | / |
| Age  Greater than or equal to 47 years old  Less than 47 years old | 18  9  9 | -1.87 [-2.53, -1.21]  -1.42 [-2.43, -0.41]  -2.36 [-3.27, -1.45] | 113.60  87.12  44.73 | 0.00  0.00  0.00 | 87.28  90.82  82.12 | 0.18 | 0.59 |
| Course of disease  Greater than or equal to four years  Less than four years | 16  8  8 | -1.75 [-2.44, -1.05]  -1.56 [-2.64, -0.49]  -1.94 [-2.95, -0.93] | 126.29  76.00  50.22 | 0.00  0.00  0.00 | 88.12  90.79  86.06 | 0.62 | 0.87 |
| Course of treatment  Greater than or equal to 12 weeks  Less than 12 weeks | 18  6  12 | -1.87 [-2.53, -1.21]  -1.63 [-2.75, -0.52]  -1.97 [-2.87, -1.08] | 113.60  37.41  95.13 | 0.00  0.00  0.00 | 87.28  86.64  88.44 | 0.64 | 0.82 |
| Experimental group  DJD  DJD + C | 18  2  16 | -1.87 [-2.53, -1.21]  -1.03 [-0.00, -2.05]  -2.13 [-2.76, -1.50] | 113.60  0.67  104.44 | 0.00  0.41  0.00 | 87.28  0.00  85.64 | 0.00 | 0.08 |
| Control group  Multiple drugs  Single drug | 18  8  10 | -1.87 [-2.53, -1.21]  -1.57 [-2.60, -0.54]  -2.15 [-2.91, -1.38] | 113.60  72.02  38.88 | 0.00  0.00  0.00 | 87.28  90.28  76.85 | 0.38 | 0.97 |
| Random sequence generation  Low risk  Unclear risk | 18  12  6 | -1.87 [-2.53, -1.21]  -1.89 [-2.65, -1.13]  -1.80 [-3.33, -0.27] | 113.60  98.04  33.88 | 0.00  0.00  0.00 | 87.28  88.78  85.24 | 0.91 | 0.95 |
| 10. Grip strength of both hands | 9 | 19.51 [14.82, 24.20] | 31.67 | 0.00 | 74.74 | / | / |
| Age  Greater than or equal to 47 years old  Less than 47 years old | 9  3  6 | 19.51 [14.82, 24.20]  13.37 [8.32, 18.42]  22.30 [18.35, 26.26] | 31.67  31.67  5.76 | 0.00  0.04  0.33 | 74.74  69.25  13.14 | 0.01 | / |
| Course of disease  Greater than or equal to four years  Less than four years | 6  4  2 | 18.36 [12.44, 24.28]  19.31 [13.93, 24.70]  16.16 [1.15, 31.17] | 21.60  5.42  6.92 | 0.00  0.14  0.01 | 76.85  44.68  85.55 | 0.70 | / |
| Course of treatment  Greater than or equal to 12 weeks  Less than 12 weeks | 9  4  5 | 19.51 [14.82, 24.20]  18.33 [10.20, 26.47]  19.86 [15.45, 24.27] | 31.67  17.87  6.01 | 0.00  0.00  0.20 | 74.74  83.21  33.44 | 0.75 | / |
| Experimental group  DJD  DJD + C | 9  1  8 | 19.51 [14.82, 24.20]  15.30 [9.54, 21.06]  20.47 [14.98, 25.97] | 31.67  0.00  31.67 | 0.00  /  0.00 | 74.74  /  77.90 | 0.20 | / |
| Control group  Multiple drugs  Single drug | 9  4  5 | 19.51 [14.82, 24.20]  15.75 [9.58, 21.91]  21.25 [17.48, 25.01] | 31.67  13.10  4.24 | 0.00  0.00  0.37 | 74.74  77.09  5.77 | 0.14 | / |
| Random sequence generation  Low risk  Unclear risk | 9  6  3 | 19.51 [14.82, 24.20]  17.01 [12.03, 21.99]  25.29 [19.13, 31.46] | 31.67  20.14  0.52 | 0.00  0.00  0.77 | 74.74  75.18  0.00 | 0.04 | / |
| 11. Visual analogue scale | 10 | -4.18 [-5.60, -2.76] | 156.65 | 0.00 | 94.25 | / | / |
| Age  Greater than or equal to 47 years old  Less than 47 years old | 10  2  8 | -4.18 [-5.60, -2.76]  -1.56 [-1.94, -1.17]  -6.80 [-9.15, -4.46] | 156.65  0.51  155.12 | 0.00  0.47  0.00 | 94.25  0.00  95.49 | 0.00 | 0.96 |
| Course of disease  Greater than or equal to four years  Less than four years | 9  3  6 | -3.44 [-4.74, -2.14]  -9.14 [-19.32, 1.05]  -1.77 [-2.77, -0.78] | 125.75  84.83  39.55 | 0.00  0.00  0.00 | 93.64  97.64  87.36 | 0.16 | 0.56 |
| Course of treatment  Greater than or equal to 12 weeks  Less than 12 weeks | 10  4  6 | -4.18 [-5.60, -2.76]  -3.70 [-5.99, -1.41]  -4.90 [-7.03, -2.77] | 156.65  37.62  116.80 | 0.00  0.00  0.00 | 94.25  92.02  95.72 | 0.45 | 0.71 |
| Experimental group  DJD  DJD + C | 10  1  9 | -4.18 [-5.60, -2.76]  -1.07 [-9.96, 7.82]  -4.26 [-5.71, -2.82] | 156.65  0.00  156.63 | 0.00  /  0.00 | 94.25  /  94.89 | 0.49 | 0.46 |
| Control group  Multiple drugs  Single drug | 10  5  5 | -4.18 [-5.60, -2.76]  -4.17 [-6.23, -2.12]  -5.08 [-7.59, -2.57] | 156.65  93.12  57.49 | 0.00  0.00  0.00 | 94.25  95.70  93.04 | 0.58 | 0.41 |
| Random sequence generation  Low risk  Unclear risk | 10  6  4 | -4.18 [-5.60, -2.76]  -3.91 [-5.46, -2.37]  -9.69 [-20.38, 0.99] | 156.65  110.38  40.11 | 0.00  0.00  0.00 | 94.25  95.47  92.52 | 0.29 | 0.50 |
| 12. Health assessment questionnaire | 9 | -0.62 [-0.83, -0.41] | 41.26 | 0.00 | 80.61 | / | / |
| Age  Greater than or equal to 47 years old  Less than 47 years old | 9  5  4 | -0.62 [-0.83, -0.41]  -0.79 [-1.06, -0.53]  -0.41 [-0.59, -0.24] | 41.26  8.49  8.63 | 0.00  0.08  0.03 | 80.61  52.90  65.25 | 0.02 | 0.33 |
| Course of disease  Greater than or equal to four years  Less than four years | 8  4  4 | -0.67 [-0.84, -0.40]  -0.50 [-0.80, -0.20]  -0.83 [-1.14, -0.53] | 40.85  80.40  5.37 | 0.13  0.00  0.15 | 82.87  80.40  44.18 | 0.13 | 0.56 |
| Course of treatment  Greater than or equal to 12 weeks  Less than 12 weeks | 9  3  6 | -0.62 [-0.83, -0.41]  -0.58 [-.081, -0.35]  -0.66 [-0.99, -0.33] | 41.26  3.43  37.62 | 0.00  0.18  0.00 | 80.61  41.62  86.71 | 0.70 | 0.38 |
| Experimental group  DJD  DJD + C | 9  0  9 | -0.62 [-0.83, -0.41]  /  -0.62 [-0.83, -0.41] | 41.26  /  41.26 | 0.00  /  0.00 | 80.61  /  80.61 | / | / |
| Control group  Multiple drugs  Single drug | 9  6  3 | -0.62 [-0.83, -0.41]  -0.69 [-1.03, -0.35]  -0.49 [-0.75, -0.23] | 41.26  33.49  6.12 | 0.00  0.00  0.05 | 80.61  85.07  67.34 | 0.36 | 0.36 |
| Random sequence generation  Low risk  Unclear risk | 9  6  3 | -0.62 [-0.83, -0.41]  -0.73 [-1.13, -0.33]  -0.58 [-0.87, -0.30] | 41.26  23.18  15.95 | 0.00  0.00  0.00 | 80.61  78.43  87.46 | 0.55 | 0.35 |
| 13. Disease activity score in 28 joints | 9 | -0.81 [-1.01, -0.61] | 21.05 | 0.01 | 62.00 | / | / |
| Age  Greater than or equal to 47 years old  Less than 47 years old | 9  4  5 | -0.81 [-1.01, -0.61]  -0.89 [-1.02, -0.77]  -0.73 [-1.24, -0.22] | 21.05  3.57  15.27 | 0.01  0.31  0.00 | 62.00  15.89  73.81 | 0.55 | 0.60 |
| Course of disease  Greater than or equal to four years  Less than four years | 9  5  4 | -0.81 [-1.01, -0.61]  -0.83 [-1.17, -0.49]  -0.81 [-1.09, -0.54] | 21.05  11.02  8.05 | 0.01  0.03  0.05 | 62.00  63.70  62.72 | 0.94 | 0.83 |
| Course of treatment  Greater than or equal to 12 weeks  Less than 12 weeks | 9  6  3 | -0.81 [-1.01, -0.61]  -0.84 [-1.09, -0.60]  -0.67 [-1.21, -0.14] | 21.05  11.98  8.05 | 0.01  0.04  0.02 | 62.00  58.26  75.14 | 0.58 | 0.89 |
| Experimental group  DJD  DJD + C | 9  2  7 | -0.81 [-1.01, -0.61]  -0.59 [-1.49, -0.30]  -0.83 [-1.05, -0.61] | 21.05  7.74  12.01 | 0.01  0.01  0.06 | 62.00  87.08  50.06 | 0.61 | 0.96 |
| Control group  Multiple drugs  Single drug | 9  6  3 | -0.81 [-1.01, -0.61]  -0.89 [-1.11, -0.66]  -0.58 [-1.12, -0.03] | 21.05  9.42  14.55 | 0.01  0.09  0.00 | 62.00  46.90  82.68 | 0.30 | 0.95 |
| Random sequence generation  Low risk  Unclear risk | 9  6  3 | -0.81 [-1.01, -0.61]  -0.82 [-0.97, -0.67]  -0.91 [-1.89, -0.07] | 21.05  8.40  12.53 | 0.01  0.14  0.00 | 62.00  40.45  84.04 | 0.86 | 0.61 |
| 14. Adverse events | 22 | -0.11 [-0.16, -0.06] | 60.88 | 0.00 | 73.88 | / | / |
| Age  Greater than or equal to 47 years old  Less than 47 years old | 22  12  10 | -0.11 [-0.16, -0.06]  -0.10 [-0.17, -0.03]  -0.12 [-0.19, -0.05] | 60.88  33.01  26.81 | 0.00  0.00  0.00 | 73.88  72.78  71.12 | 0.71 | 0.96 |
| Course of disease  Greater than or equal to four years  Less than four years | 21  10  11 | -0.11 [-0.16, -0.06]  -0.09 [-0.15, -0.03]  -0.13 [-0.22, -0.05] | 60.67  26.25  33.84 | 0.00  0.00  0.00 | 75.37  68.99  76.21 | 0.42 | 0.84 |
| Course of treatment  Greater than or equal to 12 weeks  Less than 12 weeks | 22  9  13 | -0.11 [-0.16, -0.06]  -0.07 [-0.14, -0.00]  -0.14 [-0.20, -0.08] | 60.88  20.80  39.67 | 0.00  0.01  0.00 | 73.88  60.35  75.64 | 0.15 | 0.53 |
| Experimental group  DJD  DJD + C | 22  2  20 | -0.11 [-0.16, -0.06]  -0.22 [-0.41, -0.03]  -0.10 [-0.15, -0.05] | 60.88  3.19  52.39 | 0.00  0.07  0.00 | 73.88  68.67  72.08 | 0.23 | 0.53 |
| Control group  Multiple drugs  Single drug | 22  13  9 | -0.11 [-0.16, -0.06]  -0.10 [-0.16, -0.04]  -0.12 [-0.20, -0.04] | 60.88  28.34  32.28 | 0.00  0.00  0.00 | 73.88  64.02  82.72 | 0.65 | 0.84 |
| Random sequence generation  Low risk  Unclear risk | 22  14  8 | -0.11 [-0.16, -0.06]  -0.06 [-0.10, -0.01]  -0.19 [-0.29, -0.09] | 60.88  19.51  38.43 | 0.00  0.11  0.00 | 73.88  40.63  85.60 | 0.01 | 0.11 |

**Note:** DJD: Duhuo-Jisheng decoction; C: control group. **Article title:** Efficacy and safety of Duhuo-Jisheng decoction in rheumatoid arthritis: A systematic review and meta-analysis of 42 randomized controlled trials. **First author**: Pengda Qu
