# Supplementary material for: Efficacy and safety of Duhuo-Jisheng decoction in rheumatoid arthritis: A systematic review and meta-analysis of 42 randomized controlled trials
Source: Medicine (Baltimore). 2023 Nov 3;102(44):e35513. doi: 10.1097/MD.0000000000035513 (PMC10627613; doi:10.1097/MD.0000000000035513)
Supplement: Supplementary file 4 [file medi-102-e35513-s004.docx]

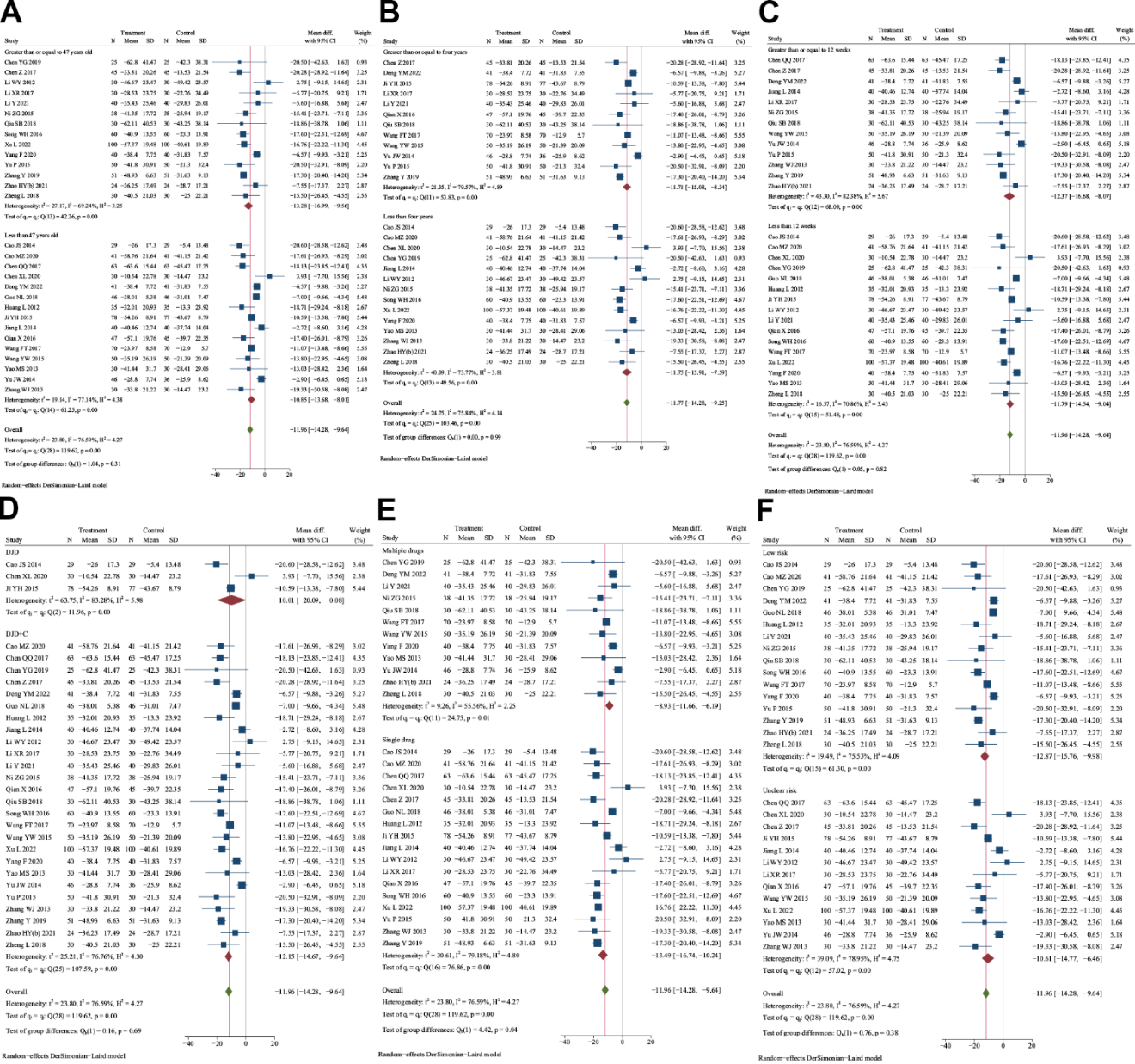
 Figure S2. Forest plot for subgroup analysis of ESR. Subgroups of different (A) ages, (B) course of disease, (C) course of treatment, (D) interventions used in the experimental group, (E) interventions used in the control group, and (F) bias assessment of random sequence generation. Article title: Efficacy and safety of Duhuo-Jisheng decoction in rheumatoid arthritis: A systematic review and meta-analysis of 42 randomized controlled trials. First author: Pengda Qu
