# Supplementary material for: Efficacy and safety of Duhuo-Jisheng decoction in rheumatoid arthritis: A systematic review and meta-analysis of 42 randomized controlled trials
Source: Medicine (Baltimore). 2023 Nov 3;102(44):e35513. doi: 10.1097/MD.0000000000035513 (PMC10627613; doi:10.1097/MD.0000000000035513)
Supplement: Supplementary file 11 [file medi-102-e35513-s011.docx]

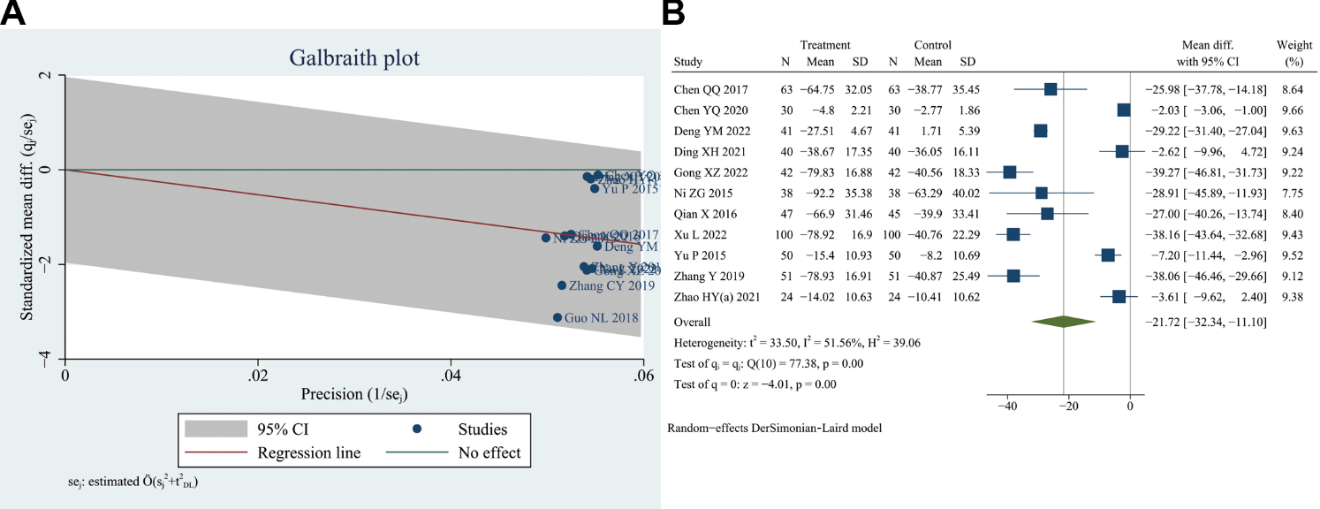
 Figure S9. Sensitivity analysis of TNF-α. (A) Galbraith plot; (B) Forest plot after excluding studies that may be the sources of heterogeneity. Article title: Efficacy and safety of Duhuo-Jisheng decoction in rheumatoid arthritis: A systematic review and meta-analysis of 42 randomized controlled trials. First author: Pengda Qu
