# Supplementary material for: Efficacy and safety of Duhuo-Jisheng decoction in rheumatoid arthritis: A systematic review and meta-analysis of 42 randomized controlled trials
Source: Medicine (Baltimore). 2023 Nov 3;102(44):e35513. doi: 10.1097/MD.0000000000035513 (PMC10627613; doi:10.1097/MD.0000000000035513)
Supplement: Supplementary file 14 [file medi-102-e35513-s014.docx]

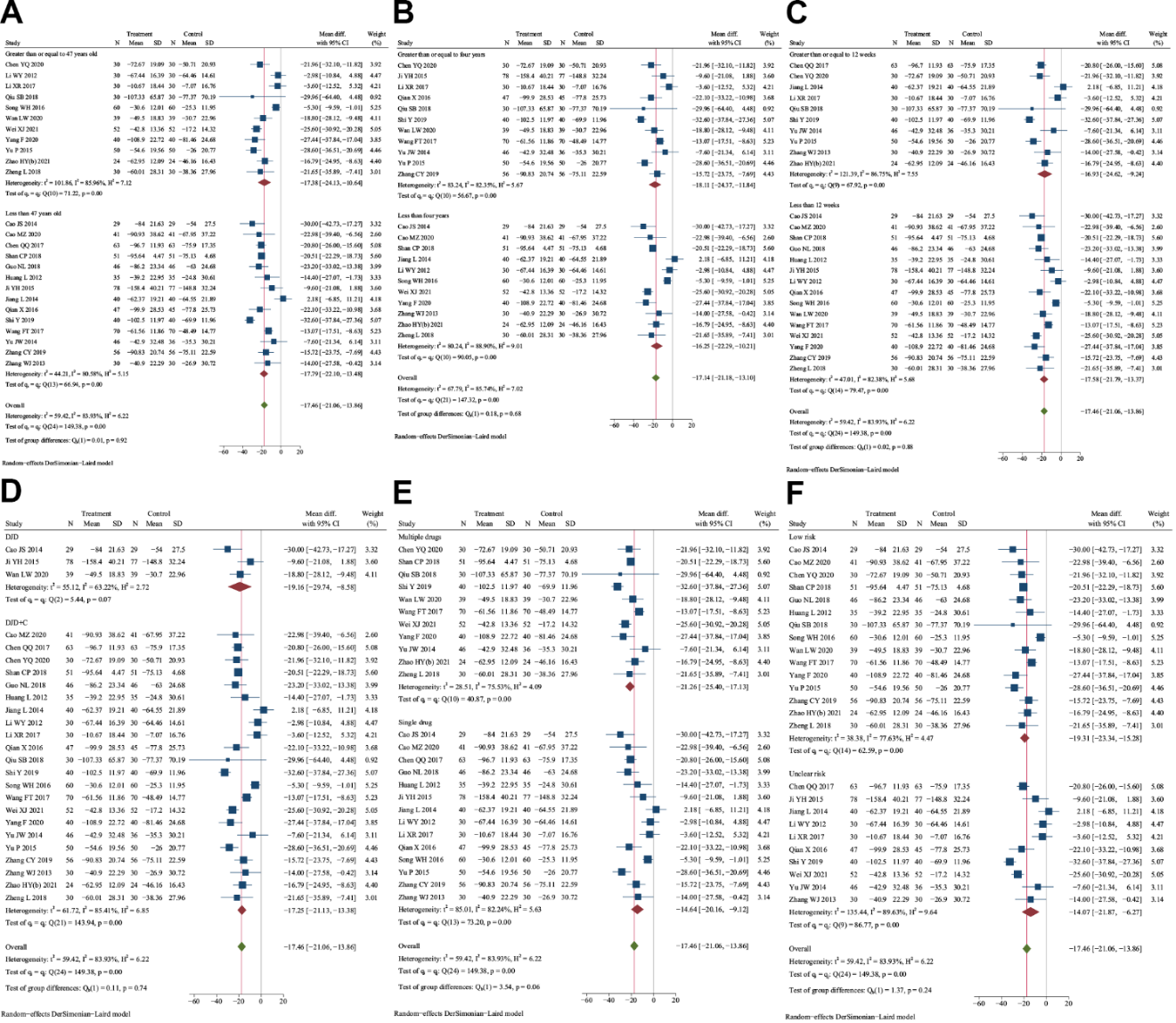
 Figure S13. Forest plot for subgroup analysis of duration of morning stiffness. Subgroups of different (A) ages, (B) course of disease, (C) course of treatment, (D) interventions used in the experimental group, (E) interventions used in the control group, and (F) bias assessment of random sequence generation. Article title: Efficacy and safety of Duhuo-Jisheng decoction in rheumatoid arthritis: A systematic review and meta-analysis of 42 randomized controlled trials. First author: Pengda Qu
